# Effect of bacteriophages on cytokine production by human peripheral blood mononuclear cells and their subpopulations

**DOI:** 10.3389/fimmu.2026.1916240

**Published:** 2026-10-13

**Authors:** Hubert Kasprzak, Monika Kniotek, Katarzyna Zielniok, Maciej Przybylski, Andrzej Górski, Ryszard Międzybrodzki

**Affiliations:** 1 Bacteriophage Laboratory, Phage Therapy Department, Hirszfeld Institute of Immunology and Experimental Therapy, Polish Academy of Sciences, Wrocław, Poland; 2 Department of Clinical Immunology, Medical University of Warsaw, Warsaw, Poland; 3 Laboratory of Cellular and Genetic Therapies, Center for Preclinical Research, Medical University of Warsaw, Warsaw, Poland; 4 Chair and Department of Medical Microbiology, Medical University of Warsaw, Warsaw, Poland; 5 Phage Therapy Unit, Hirszfeld Institute of Immunology and Experimental Therapy, Polish Academy of Sciences, Wrocław, Poland; 6 Professor Emeritus, Department of Clinical Immunology, Medical University of Warsaw, Warsaw, Poland

**Keywords:** bacteriophages, immunomodulation, lymphocytes, monocytes, PBMCs, phage therapy, pro- and anti-inflammatory cytokines

## Abstract

**Introduction:**

Fulfilling a vision that extends beyond their role as antibiotic alternatives, bacteriophages are now recognized as active immunomodulators, a concept aligning with early hypotheses of their biological activity. In addition to their bactericidal action, these properties require detailed investigation. This study aims to characterize the immunomodulatory effects of bacteriophages on pro- and anti-inflammatory cytokine production by human immune cells isolated from healthy blood donors.

**Methods:**

We assessed the secretion of a comprehensive panel of 11 cytokines (including IFN-γ, IL-6, TNF-α, and IL-10) by peripheral blood mononuclear cells and their isolated subpopulations (B and T lymphocytes, helper and cytotoxic T lymphocytes, natural killer cells, and monocytes). The cells were incubated for 24 hours with purified *Escherichia coli* phage T4 and therapeutic staphylococcal phage A5/80. Reference cells were incubated with lipopolysaccharide from *E. coli* B at a concentration corresponding to its activity in the applied phage preparations. For comparison, we also assessed cytokine responses to human adenovirus 5, used as a benchmark for a virus capable of infecting human cells.

**Results:**

Cell-specific analysis revealed a distinct hierarchy in immune responsiveness: our data suggest that monocytes may act as the primary sensors driving cytokine signatures, while we did not confirm a discernible direct response in B, natural killer, and cytotoxic T cells. Importantly, the magnitude of these responses was markedly lower than the robust inflammatory cytokine secretion triggered by human adenovirus 5, providing context for the phage-induced effects relative to a viral infection of human cells and underscoring the limited inflammatory footprint of therapeutic phages.

**Conclusion:**

These results improve our understanding of phage–human immune system interactions, which is important for appreciating their broader impact on human homeostasis and for assessing the safety of phage therapy.

## 1 Introduction

Fulfilling a vision that extends beyond their role as antibiotic alternatives, bacteriophages are now also recognized as active immunomodulators (1, 2), a concept that aligns with their initial discovery as agents of immunity by Félix d’Hérelle (3). This marks a significant evolution from the long-held assumption that phages are biologically inert toward mammalian systems to a recognition of their capacity for direct interaction with eukaryotic cells (1, 4–6). This paradigm shift paradoxically returns to d’Hérelle’s earliest hypotheses. Although he ultimately concluded that must be independent, parasitic “ultramicrobes”, his initial intuition regarding their interaction with the host has found new resonance (3). Consequently, the field must now move beyond broad concepts of immunomodulation to a precise mechanistic characterization of these direct cellular responses (7).

Bacteriophages interact with the epithelial cells that form the mucosal barrier (8). These cells express a broad range of pattern recognition receptors (PRRs) and possess significant immune-regulatory functions, including those responsive to viral detection (9). Going beyond theoretical models, experimental studies indicate that phages can directly modulate epithelial cell responses. Specifically, Borysowski et al. (10) demonstrated that phage exposure significantly alters the expression of genes involved in antimicrobial immunity, including those encoding cytokines and Toll-like receptors (TLRs). Furthermore, following administration, this interaction may not be merely localized because phages can translocate, for example, from the gut to the bloodstream and distribute to various tissues throughout the body (11, 12). This systemic presence necessitates a precise analysis of their direct cellular impact to ensure therapeutic safety (11). Recent findings identify epithelial and endothelial cell layers as a major, previously unquantified “sink” for administered phages (13). This phenomenon highlights the profound extent of phage-host cellular interactions, which affects not only pharmacokinetics but also potentially systemic immune homeostasis.

Dendritic cells (DCs) serve as professional antigen-presenting cells (APCs) and critical mediators of antiviral immunity (14). Bacteriophages are efficiently internalized by these cells through endocytosis, a process that can drive distinct immune outcomes (15). A primary mechanism for this activation involves the recognition of phage genomic DNA by the intracellular receptor TLR9. This interaction triggers a potent pro-inflammatory cascade, often characterized by the induction of IFN-γ-mediated pathways. Crucially, this activation is directly induced by the phage particles themselves and is not attributable to bacterial contaminants. For example, the contribution of residual endotoxins was functionally excluded by demonstrating that immune activation persisted in DCs lacking CD14, a co-receptor essential for lipopolysaccharide (LPS) recognition (15). Conversely, the immune outcome is not invariably inflammatory. Phagocytosis of phages by healthy human peripheral blood monocytes has been shown to induce a “tolerogenic” state, characterized by the downregulation of their key surface markers such as CD14 and CD86 (16). This non-inflammatory profile has been attributed to capsid stability; studies utilizing highly purified, intact T4 phages demonstrate that they can be internalized without activating cytosolic DNA-sensing pathways like TLR9 or cGAS-STING, suggesting that the phage genomic DNA remains sequestered within the capsid (17). Furthermore, beyond mere immune evasion, this internalization can activate cellular growth and survival pathways, promoting the hypothesis that eukaryotic cells may even utilize bacteriophages as a metabolic resource to support their own proliferation (17).

Exposure of human peripheral blood mononuclear cells (PBMCs) to phages results in a complex cytokine profile, distinguishing phage action from that of conventional pathogens (18, 19). Phages can induce key pro-inflammatory cytokines, such as IL-6, TNF-α, and IL-1β, which initiate an innate immune response (18, 20). However, this activation requires careful regulation, as excessive phage-induced IFN-γ has been linked to exacerbated intestinal inflammation (15). Crucially, this pro-inflammatory signaling is often counterbalanced. Some phages actively promote the expression of anti-inflammatory mediators, including IL-10 and the IL-1 receptor antagonist (19). In other instances, phages exert an anti-inflammatory effect by directly suppressing the production of pro-inflammatory cytokines, such as IL-1β, or by dampening excessive inflammatory responses to a bacterial challenge (11, 20, 21). This dual capacity to both trigger and modulate inflammation suggests that some phages possess a homeostatic potential, steering the host’s response toward tolerance rather than unconstrained pathology. While phages are increasingly recognized as diverse modulators of the host immune system, the specific factors driving their differential immunogenicity remain largely unknown (14). Current understanding of these interactions relies heavily on data derived from bulk PBMC populations or immortalized cell lines. This methodological constraint inherently masks the distinct contributions of specific immune subsets. To address this gap, our study performs a cell-specific analysis of cytokine secretion to functionally delineate these varied immune responses.

Previous studies utilizing transcriptional profiling of bulk PBMCs have demonstrated a generalized anti-inflammatory gene signature induced by bacteriophages (19). However, relying on bulk populations may mask the distinct contributions of specific immune subsets, and the functional translation of these transcriptional signals into secreted proteins remains largely uncharacterized. Furthermore, biological heterogeneity in primary immune responsiveness among healthy donors can heavily influence aggregated mean results, necessitating a more granular approach.

Accordingly, our exploratory study aimed to dissect the cell-specific dynamics of cytokine secretion across individual immune subsets while accounting for natural donor heterogeneity. To the best of our knowledge, this represents the first comprehensive attempt to map phage-immune interactions using such a broad panel of PBMC subpopulations isolated from multiple independent human donors. By exposing these cells to two distinct phages at concentrations corresponding to clinically relevant therapeutic thresholds, we sought to characterize their responses at high resolution (22). Ultimately, understanding this functional translation at the cell-specific level is essential for defining the impact of bacteriophages on systemic human immune homeostasis. Characterizing these phage-specific immunological footprints could inform future medical practice by guiding the targeted selection of phage preparations that, in addition to targeting the relevant bacteria, could help induce or support a desired immune state.

## 2 Materials and methods

### 2.1 Phage preparation

This study utilized two bacteriophages: *E. coli* phage T4, obtained from the American Type Culture Collection (ATCC; USA), and staphylococcal phage A5/80, from the therapeutic phage collection of the Hirszfeld Institute of Immunology and Experimental Therapy of the Polish Academy of Sciences (HI) in Wrocław, Poland (23).

Bacterial strains for phage propagation (*E. coli* B for T4 and *Staphylococcus aureus* 80 for A5/80) were sourced from the Polish Collection of Microorganisms at the HI. Crude phage lysates were produced based on a modified protocol by Ślopek et al. (24). Specifically, the bacteriophages and their respective host bacteria were co-cultured in peptone water at 37 °C. The incubation was carried out for 8 to 16 hours until complete bacterial lysis was observed. Following this step, the resulting suspensions were clarified by filtration through a 0.22-μm filter (Millipore, Darmstadt, Germany) to remove any remaining cells.

The clarified lysates were then purified according to a protocol developed at HI (25, 26), using specific process parameters. Briefly, phage lysates were concentrated using the cross-flow membrane filtration technique with a Vivaflow 200 ultrafiltration set with a 30,000 MWCO Hydrosart membrane (Sartorius Stedim Lab Ltd., Stonehouse, UK), operating at a pressure of 3 bar. Next, phages were purified by size exclusion chromatography. The stationary phase in the 26/100 chromatographic column (GE Healthcare Life Sciences, Uppsala, Sweden) was Sepharose CL-4B (Cytiva, Uppsala, Sweden), comprising 4% cross-linked agarose with a particle size of 45–165 µm. The process was conducted at 22 °C with a 25 mL sample volume, using 0.068 M phosphate buffer (pH 7.2) as the eluent at a flow rate of 1.3 mL/min, and monitored by UV detection (Knauer, Berlin, Germany) at 280 nm. Based on the manufacturer’s specifications, the fractionation range of Sepharose CL-4B (60 kDa to 20 MDa) facilitates the elimination of low-molecular-weight host contaminants, including the highly immunogenic staphylococcal protein A (~42 kDa) (27). For phage T4, an additional affinity chromatography step was performed using a Cellufine ET clean S column (JNC Corporation, Tokyo, Japan) to remove excess bacterial endotoxin. This step was methodologically imperative to equalize the starting backgrounds; because T4 is propagated in a Gram-negative host, its crude lysate contains massive native endotoxin loads, whereas the A5/80 propagated in a Gram-positive host contains only trace environmental endotoxin. A 20 mL sample was applied to the column, which was operated at a constant flow rate of 0.8 mL/min at 22 °C. Adsorption was carried out in 0.01 M phosphate buffer (pH 7.6), followed by elution using the same buffer supplemented with 1 M NaCl. The entire separation process was continuously monitored via UV detection at 280 nm.

Upon completion of their respective purification protocols, both phage preparations were dialyzed against Ca^2+^ and Mg^2+^ free phosphate-buffered saline (CMF-PBS) and subsequently stored at 4 °C. Stock preparations of both phages were stored at 2 °C - 8 °C at a concentration of 3 (± 2) × 10^9^ PFU/mL and used as working solutions for the high phage dose. Immediately before the experiment, they were diluted tenfold in CMF-PBS to create working solutions for the low phage dose. Phage titers were determined after purification by the double agar overlay plaque assay as described by Adams (28) and were monitored throughout the study. Endotoxin activity was quantified directly in the final purified phage preparations using a chromogenic LAL assay (QCL-1000, Lonza, Switzerland), which confirmed an endotoxin activity of 10 ± 3 EU/mL in both phage stock preparations.

### 2.2 Adenovirus preparation

Human adenovirus 5 (AdV-5; ATCC VR-5) was propagated in A549 cells (ATCC CCL-185). After 48 h of incubation, intracellular virions were released by repeated freeze–thaw cycles. The virus suspension was then clarified by centrifugation and filtered through a 0.8 µm filter. To achieve the high viral titers required for subsequent assays, the clarified virus-containing suspension was adjusted to 0.45 M NaCl and 0.1 mg/L dextran sodium salt, followed by centrifugation at 15,000 rpm for 60 min at 4 °C. After removal of the supernatant, the pellet was resuspended in 50 µL of phosphate-buffered saline (PBS). The resulting concentrated virus preparation was then titrated using the Reed–Muench method according to a previously described protocol (25, 29) and stored in the vapor phase of liquid nitrogen at a titer of 5 × 10^9^ TCID_50_/mL. Contamination of the AdV-5 by endotoxin was tested with the use of of a chromogenic LAL test (GenScript, Piscataway, NJ, USA). The undiluted AdV-5 stock was used as the working solution for the high viral dose, whereas its tenfold dilution in Roswell Park Memorial Institute (RPMI) 1640 medium was used as the working solution for a low viral dose. To estimate the AdV-5 titer in PFU/mL from the TCID_50_ value, we applied a conversion factor of 0.7 based on the Poisson distribution (30).

### 2.3 Lipopolysaccharide preparation

*E. coli* B lipopolysaccharide (LPS) was prepared from a bacterial culture grown for 24 h at 37 °C in liquid LB medium (pH 7.0) using a 10 L BioFlo415 fermenter (Eppendorf, Germany), following the procedure described by Kaszowska et al. (31). After cultivation, the bacteria were killed with 0.5% phenol and harvested by centrifugation at 39,000 rpm using a CEPA LE flow centrifuge (New Brunswick Scientific, USA) (32). The resulting bacterial mass was washed three times with distilled water and lyophilized. Lipopolysaccharide was subsequently isolated from the dried mass using the phenol/chloroform/petroleum ether (PCP) method and purified by ultracentrifugation at 105,000 × *g* for 4 h at 4 °C (33). The purified LPS was then diluted in CMF-PBS and dispersed with a Sonopuls HD 2070 ultrasonic homogenizer (Bandelin, Germany). The final stock solution, which had an endotoxin activity of 5000 EU/mL as measured by the LAL test (Thermo Fisher Scientific, Waltham, MA, USA) as it was done for the phages, was aliquoted into Eppendorf tubes and frozen at -20 °C for storage. Immediately before the experiments, an aliquot was thawed and appropriately diluted in CMF-PBS to prepare working suspensions (10 EU/mL and 1 EU/mL).

### 2.4 Isolation of PBMCs and their subpopulations

Peripheral blood mononuclear cells were isolated from buffy coats obtained from healthy blood donors. As an additional immunological exclusion criterion, any donor exhibiting an inverted CD4/CD8 ratio during initial flow cytometry screening was automatically excluded from the study prior to any downstream processing. The isolation utilized density gradient centrifugation. Briefly, the buffy coat (∼20 mL) was diluted approximately 1:1 with PBS and layered onto Histopaque 1077 (Sigma-Aldrich, St. Louis, MO, USA). The gradient was then centrifuged at 650×g for 20 min (with deceleration off) at room temperature to separate the PBMC interface. The collected PBMCs were washed three times in PBS. The first two washes were performed at 304 × *g* for 8 min, and the final wash at 180×g for 10 min, with all procedures carried out at room temperature. Their post-isolation purity was verified by bright-field light microscopy based on cell morphology and/or flow cytometry based on CD45 versus SSC analysis. After the final wash, the PBMC pool was split into two aliquots. The first aliquot, designated for direct *ex vivo* stimulation, was resuspended in RPMI 1640 medium containing HEPES and 2 mM L-glutamine (Gibco, UK) supplemented with 20% human AB serum (Sigma-Aldrich, St. Louis, MO, USA). The second aliquot, designated for the isolation of specific immune subpopulations, was resuspended in MojoSort buffer. PBMC subpopulations, including T and B lymphocytes, T-helper cells, cytotoxic T cells, natural killer (NK) cells, and monocytes, were subsequently isolated from this second fraction by negative magnetic selection using the MojoSort system (BioLegend, San Diego, CA; cat. No. #480021, #480009, #480011, #480053, #480081, and #480059). The separation was performed according to the manufacturer’s protocols, using 10^7^ PBMCs prepared in 100 µL of MojoSort buffer per tube. The desired populations were collected as the untouched, unlabeled liquid fraction and resuspended in the same medium formulation as PBMCs. Cellular viability and total cell counts were assessed utilizing the trypan blue exclusion method in a Bürker chamber under a light microscope; viability consistently exceeded 90% across all isolated fractions.

### 2.5 Phenotypic characterization of isolated cell populations and gating strategy for their purity assessment

To estimate the number of PBMCs needed to obtain the desired number of cells of a given subpopulation, surface marker staining was performed on PBMCs isolated from each donor. Briefly, 10^5^ cells were resuspended in 100 µL of Dulbecco′s Phosphate Buffered Saline (DPBS; Corning, USA) and incubated with a cocktail of titrated antibodies for 15 min at room temperature in the dark. The antibody cocktail included single fluorochrome-conjugated antibodies against CD45, CD3, CD4, CD8, CD56, CD19, CD16, CD14, and CD33 (all from Beckman Coulter). The antibody volumes and fluorochromes used are listed in **Table 1**. After staining, the cells were washed with 2 mL of PBS by centrifugation (450 × *g* for 5 min at room temperature) and resuspended in 100 µL of PBS for acquisition. The same procedure was applied to label the isolated PBMC subpopulations to assess their purity.

**Table 1:** Antibodies used for lymphocyte subpopulation staining (clone and volume).

Target antigen	Fluorochrome	Clone	Isotype	Volume*	Manufacturer	Catalog no.
CD3	APC-ALEXA FLUOR 750	UCHT1	IgG1, mouse	1 µl	Beckman Coulter	A94680
CD4	APC	13B8	IgG1, mouse	0,5 µl	Beckman Coulter	IM2468
CD8	APC-ALEXA FLUOR-700	B9.11	IgG1, mouse	0,5 µl	Beckman Coulter	B49181
CD14	FITC	RMO52	IgG2a, mouse	1 µl	Beckman Coulter	B36297
CD33	PC7	D3HL60.251	IgG1, mouse	0,5 µl	Beckman Coulter	B92408
CD45	KO525	J33	IgG1, mouse	2 µl	Beckman Coulter	B36294
CD16	PE	3G8	IgG1, mouse	0,5 µl	Beckman Coulter	A07766
CD56	PC5.5	N901	IgG1, mouse	0,5 µl	Beckman Coulter	B49189
CD19	ECD	J3-119	IgG1, mouse	0,5 µl	Beckman Coulter	A07770

* per 1×10^5^ cells in 100 µL of DPBS.

Before the first use, all antibodies were titrated on PBMCs by serial dilution to determine the optimal staining concentration. The dilution yielding the best signal-to-noise ratio was selected for subsequent experiments.

Samples were acquired on a DxFLEX flow cytometer (Beckman Coulter, Inc., USA) equipped with three excitation lasers: 405 nm (violet, 8 fluorescence detectors), 488 nm (blue, 5 fluorescence detectors), and 638 nm (red, 3 fluorescence detectors). Instrument settings and fluorescence detector voltages were optimized using dedicated Daily QC Fluorospheres (Beckman Coulter, USA). Forward scatter (FSC) was collected from the blue laser and side scatter (SSC) from the violet laser. A minimum of 10,000 events were collected per sample within the lymphocyte gate. Data were acquired using dedicated CytExpert software and analyzed offline with Kaluza Analysis software (both from Beckman Coulter, USA). Fluorescence compensation was set using PBMCs. For each marker, stained cells and the corresponding unstained control were acquired on the DxFLEX cytometer, and the compensation matrices were calculated automatically in CytExpert and manually adjusted if necessary.

Cells were gated based on their morphological properties using FSC and SSC. Subsequently, doublets were excluded from the analysis using an FSC-height (FSC-H) versus FSC-area (FSC-A) plot. From the resulting single-cell population, the total leukocyte fraction was identified based on the high expression of the pan-leukocyte marker CD45 plotted against side scatter (SSC vs. CD45-KO525). The immunophenotype and purity of the isolated cell fractions were evaluated exclusively within the designated CD45^+^ leukocyte gate. Lineage-specific surface markers were used to define T lymphocytes as CD3^+^, T helper (Th) cells as CD4^+^, cytotoxic T (Tc) cells as CD8^+^, B lymphocytes as CD19^+^, pan-monocytes as CD14^+^ and/or CD16^+^, and NK cells as CD3^-^CD56^+^. Visual representations of this detailed gating strategy and representative dot plots are provided in **Supplementary Figure S1**.

### 2.6 *In vitro* experiments

Before the experiments, the isolated cell suspension was adjusted to 2 × 10^6^ cells/mL using RPMI 1640 containing HEPES and 2 mM L-glutamine supplemented with 20% human AB serum. Cells were then seeded at 100 μL per well into 96-well U-bottom cell culture plates (SPL Life Sciences, Pocheon, Republic of Korea). Subsequently, 80 μL of RPMI 1640 containing HEPES and 2 mM L-glutamine and 20 μL of the appropriate phage suspension were added, resulting in a final culture volume of 200 μL per well. This yielded two final phage doses for the experiments: a high dose of 3 (± 2) × 10^8^ PFU/mL and a low dose of 3 (± 2) × 10^7^ PFU/mL. Control cells were treated with 20 μL of LPS suspension (corresponding to a final activity of 1 EU/mL in the high-dose setup and 0.1 EU/mL in the low-dose setup), whereas unstimulated cells received 20 μL CMF-PBS instead of phages. For comparative viral challenge experiments performed with PBMCs, the cells were additionally exposed to 20 μL of the virus suspension at two final doses: a high virus dose of 5 × 10^8^ TCID_50_/mL and a low virus dose of 5 × 10^7^ TCID_50_/mL. To maintain the same final volume of 200 μL per well, the volume of RPMI 1640 medium added to the virus-treated wells was reduced from 80 μL to 60 μL. All treatments were performed in triplicate or quadruplicate. After 24 h of incubation at 37 °C in a humidified atmosphere containing 5% CO_2_, the plates were centrifuged, and 120 μL of supernatant from each corresponding well was pooled into a single 1.5 mL microcentrifuge tube and frozen (≤ −20 °C) until analysis.

### 2.7 Cytokine quantification by Luminex Assay

Cytokine concentration was quantified in cell supernatants using multiplex bead-based immunoassays (Luminex^®^ Assays, R&D Systems, Bio-Techne, Minneapolis, MN, USA), according to the manufacturer’s protocols. A total of eleven cytokines were measured using two distinct kits:

1. Human High Sensitivity Cytokine Premixed Kit B (Performance Assay, Catalog No. FCSTM14) was used to quantify IFN-γ, TNF-α, IL-1β, IL-2, IL-5, IL-6, IL-13, and IL-17A;
2. Human Premixed Multi-Analyte Kit (Discovery Assay, Catalog No. LXSAHM) was used to quantify IL-4, IL-10, and IL-21.

The experiment involved the use of magnetic microparticles, each characterized by a distinct internal fluorophore ratio and coated with analyte-specific capture antibodies. Briefly, undiluted supernatant samples and cytokine standards were assayed in duplicate on 96-well microplates. Procedural details varied depending on the kit used: the High Sensitivity Kit (FCSTM14) required a 100 μL sample volume per well and a 3-hour microparticle incubation, whereas the Discovery Kit (LXSAHM) utilized a 50 μL sample volume and a 2-hour incubation. All incubation steps were conducted on an orbital shaker set at 800 ± 50 rpm. After washing, biotinylated detection antibodies were added, followed by a streptavidin-phycoerythrin (SA-PE) to complete the sandwich complex, with PE fluorescence proportional to analyte concentration. Microparticle classification and reporter fluorescence (MFI) were detected using a two-laser system, while cytokine concentrations were calculated from standard curves fitted to a 5-PL model according to the manufacturer’s instructions. The lower limit of quantification (LLQ) for each analyte was defined as the concentration of the lowest standard included in the assay. The mean LLQs calculated for the respective assays are shown in the figures. Measurements were performed on a Luminex 200 flow-based platform (Luminex Corp., Austin, Texas, USA) and data were analyzed using Belysa 1.1.0 Software (Merck Millipore, Catalog No. 40-122).

### 2.8 Statistical analysis

Due to the exploratory nature of this study, an *a priori* statistical power calculation was not performed. To optimize resource allocation while addressing natural donor heterogeneity, this study was prospectively designed as an exploratory, hypothesis-generating screening. We employed a deliberate, adaptive strategy where the predefined biological decision rule for cohort expansion was based on initial target cell responsiveness, prioritizing biological effect size independent of formal statistical hypothesis testing (34, 35). Initial cohorts (n = 3-4) were analyzed across all subpopulations to establish baseline reactivity. For cell subpopulations that did not exhibit any consistent tendency to react specifically to the phage suspension, further recruitment was capped. Conversely, for subpopulations identified as primary responders, the cohorts were expanded (n = 7-9) to characterize their distinct cytokine signatures. Therefore, the analytical approach was stratified based on the sample size of the isolated subpopulations:

- Descriptive statistics were used for subpopulations with a limited sample size analyzed only during the initial screening (n = 3-4, comprising T lymphocytes, cytotoxic T cells, B lymphocytes, and NK cells). For these groups, data are reported solely using descriptive statistics to illustrate general response trends.
- Inferential statistics were performed for cell populations with a sufficient sample size obtained during the further recruitment phase (n > 5, comprising total PBMCs, monocytes, and T helper cells). Due to the observed high inter-donor variability, differences in cytokine secretion levels between phage preparation-stimulated cells and their corresponding matched controls were evaluated using the non-parametric two-sided exact Wilcoxon signed-rank test for paired samples. To control for planned multiple comparisons, the exact *p*-values (*p*_exact_) were adjusted using the Benjamini-Hochberg false discovery rate (FDR) procedure to yield adjusted p-values (*p*_adj_). For these analyses, a *p_adj_* < 0.05 was considered statistically significant. Cases where only *p*_exact_ < 0.05 were described as trends. This designation was applied to mitigate the risk of Type II errors; relying exclusively on stringent multiplicity corrections in such early-phase *ex vivo* screening can disproportionately inflate this error rate, potentially obscuring true biological effects (36–38). Furthermore, to provide a robust statistical evaluation independent of arbitrary significance thresholds, we calculated the Hodges-Lehmann estimates (HL) of the location shift together with their 95% confidence intervals. This non parametric approach quantifies the absolute magnitude of the difference (effect size) and estimation uncertainty, which is critical for objectively evaluating biological relevance (37, 38). The complete set of descriptive and inferential statistics—including sample sizes, median values with data ranges, exact and adjusted *p*-values, and effect size estimates—is detailed in **Supplementary Tables S1**-**S3**. Statistics for IL-5 are not reported because its concentrations consistently fell below the LLQ. Statistical analyses were performed using the R programming environment version 4.6.1 (R Foundation for Statistical Computing, Vienna, Austria). Data visualizations were generated using GraphPad Prism version 10.6.1 (GraphPad Software, San Diego, CA, USA) and shown as floating bars on a logarithmic scale.

Across all experiments, data are visually presented as floating bars on a logarithmic scale to capture the full range of biological observations. The internal horizontal line indicates the median value, while the upper and lower boundaries of the bar represent the absolute maximum and minimum values, respectively.

## 3 Results

- Cellular response patterns: In PBMC cultures, phage exposure modulated adaptive and regulatory cytokine secretion. Subsequent analysis of isolated immune subsets revealed a distinct reactivity hierarchy: monocyte- and T helper cell-enriched fractions exhibited pronounced, phage-driven secretory profiles, whereas isolated B, NK, and cytotoxic T cells did not mount a distinct response under direct extracellular challenge.
- Phage-specific modulatory signatures: The evaluated phage preparations elicited distinct secretory responses rather than acting as uniform pro-inflammatory triggers. Exposure to the *E. coli* T4 phage was associated with increased IL-10 secretion in monocyte-enriched cultures, whereas the staphylococcal A5/80 preparation significantly upregulated IFN-γ in PBMC cultures and promoted a Th1-biased profile in T helper cells. This modulation was accompanied by a consistent absence of Th2-associated IL-5 secretion.

### 3.1 Experimental model and donor variability

Both studied bacteriophages have been completely sequenced and extensively characterized genomically. The therapeutic staphylococcal phage A5/80 (sequenced as clone A5W; GenBank Accession No. EU418428) is an obligate lytic myovirus currently classified within the genus *Kayvirus* (subfamily *Twortvirinae*, family *Herelleviridae*). Comprehensive genomic screening has confirmed the complete absence of lysogeny-associated genes, known bacterial virulence determinants, human-pathogenic toxins, and antimicrobial-resistance genes (23). Similarly, the model *E. coli* phage T4 (GenBank Accession No. NC_000866) is a strictly virulent myovirus, belonging to the genus *Tequatrovirus* (family *Straboviridae*), naturally devoid of lysogeny modules and pathogenic determinants. Furthermore, the immunomodulatory properties of both phages, including their interactions with mammalian cells and eukaryotic viruses, have been extensively investigated by our research group (10, 11, 25, 39–44).

To evaluate direct interactions between phages and distinct immune populations, we established an *ex vivo* model utilizing negative magnetic selection. This strategy was chosen to preserve the functional integrity and quiescent state of freshly isolated human cells. Although positive selection can yield marginally higher purity, it requires binding antibodies directly to the target cells. This cross-linking of surface receptors (e.g., CD14, CD3) inadvertently triggers intracellular signaling, leading to artificial cell activation prior to phage exposure (45). By avoiding this methodological artifact, we prioritized physiological relevance over absolute purity, ensuring that the observed cytokine profiles were attributable solely to the experimental stimuli.

The experimental design limited processing to a maximum of three distinct cell populations per donor buffy coat, thereby preserving optimal cell viability and minimizing *ex vivo* pre-activation artifacts. Importantly, every isolated culture was subjected to flow cytometry-based quality control prior to downstream assays. Finally, only subsets with confirmed high purity were subjected to multiplex cytokine quantification (**Supplementary Figure S1**). Specifically, the purity of total PBMC fractions was consistently maintained above 95%. For negatively selected subsets, purity of monocyte and B cell fractions fell within the 85-91% range, while purity of all other lymphocyte subpopulations (T, Th, Tc, and NK cells) consistently ranged between 92–98% in accepted samples. Experimental runs derived from separations falling below these criteria were excluded from further evaluation. Following the initial screening detailed in the Statistical analysis section, cohorts were expanded specifically for PBMCs, monocytes, and T helper cells. The combination of strict purity requirements and targeted cohort expansion yielded the following final sample sizes for the aggregated data: PBMCs (n = 9), monocytes (n = 7), T cells (n = 3), Th cells (n = 7), Tc cells (n = 3), B cells (n = 3), and NK cells (n = 3-4).

As expected for primary human cell models, we observed significant biological heterogeneity among the healthy donors. Specifically, the response of control cells stimulated with LPS varied considerably between individuals across different cytokine panels (**Supplementary Figures S2**, **S3**). Analyzing well-defined, isolated subsets allows for a clear distinction between the immunomodulatory profile of the phage preparations and natural donor variability, which might otherwise be masked in aggregated bulk analyses. To ensure the clinical relevance of our *ex vivo* model, we tested final phage concentrations that directly correspond to the formulation standards utilized in human applications (22). The low phage dose used in our assays reflects the therapeutic thresholds for local administration (ca. 10^7^ PFU/mL), while the high dose corresponds to those for systemic applications (ca. 10^8^ PFU/mL). Importantly, the reference baseline in our assay was established using endotoxin concentrations of 1 EU/mL and 0.1 EU/mL, matching those present in the tested phage preparations. They are close to 0.25 EU/mL, which is the defined European Pharmacopoeia limit for purified water (46).

### 3.2 PBMC cytokine secretion profiles

The cytokine secretion profiles of PBMCs following a 24-hour *ex vivo* stimulation with phages are presented in **Figure 1**. The data, derived from 7–9 healthy blood donors, revealed inter-donor variability, characteristic of primary immune responses. Nevertheless, unstimulated cells consistently maintained basal cytokine secretion levels across all measured panels, confirming their quiescent state prior to experimental challenge.

**Figure 1:**
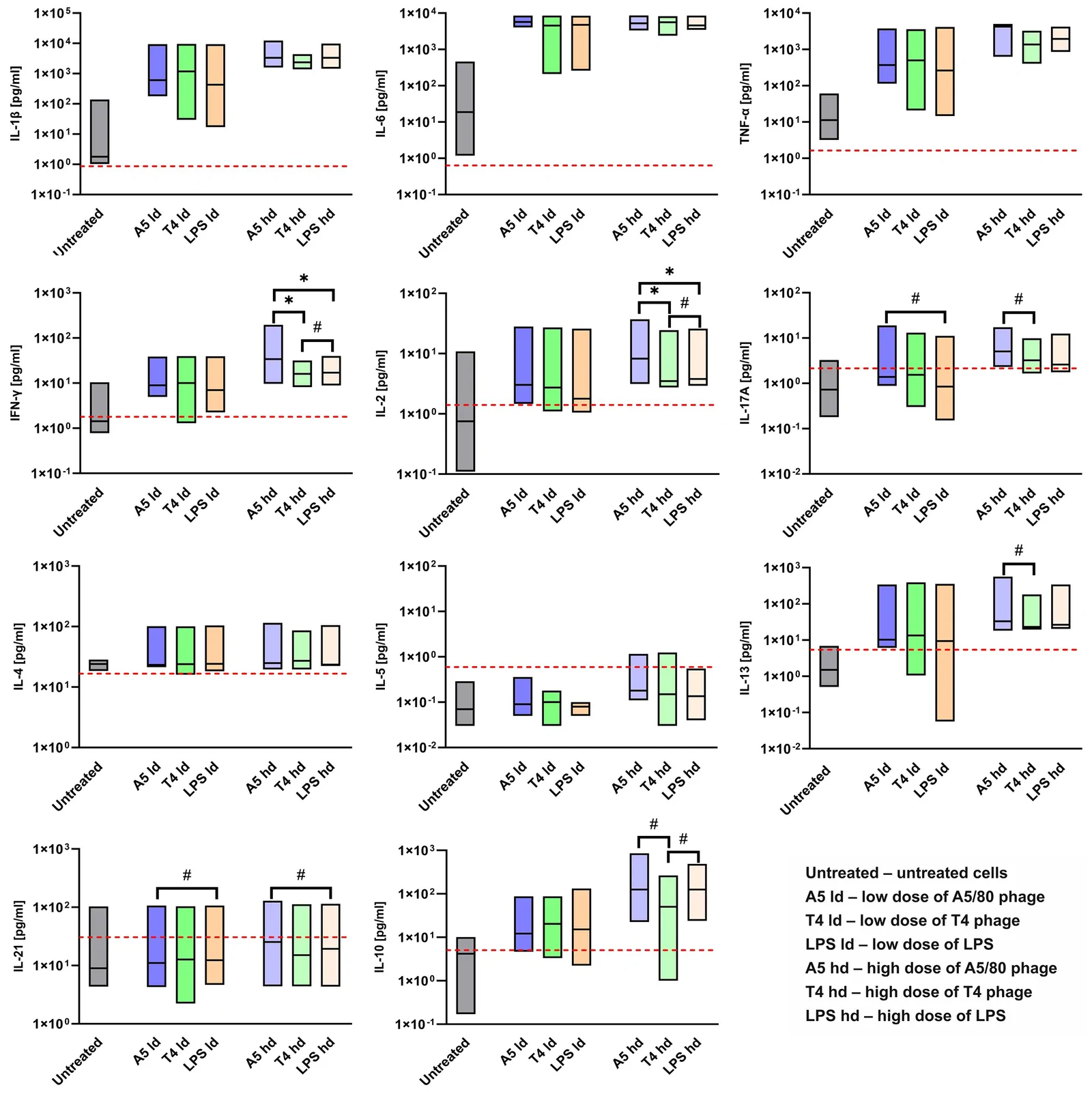
Cytokine secretion profiles of peripheral blood mononuclear cells. The internal horizontal line indicates the median value, while the upper and lower boundaries of the bar represent the absolute maximum and minimum values, respectively. Brackets mark statistical significance: ^#^denotes a nominal trend (*p_exact_* < 0.05); *indicates significant difference (*p_adj_* < 0.05) after BH-FDR adjustment. The dashed red line indicates the assay-specific lower limit of quantification.

#### 3.2.1 Pro-inflammatory and innate responses

A strong induction of the major pro-inflammatory cytokines IL-6, TNF-α, and IL-1β was observed across all stimulation conditions. For these markers, the secretion profiles induced by both A5/80 and T4 phages generally mirrored those of their respective controls. While IL-1β and TNF-α exhibited a dose-dependent increase, the high secretion of IL-6 rapidly reached the upper limit of the assay quantification range across both tested doses. This suggests that within the mixed PBMC population, the TLR4-mediated signaling triggered by residual endotoxin likely saturates the innate response, potentially masking specific phage-mediated modulation of these cytokines, a phenomenon previously described for PBMC (19) and monocytes (39). However, as demonstrated in subsequent sections, analyzing isolated responder populations (e.g., monocytes) at lower stimulus concentrations successfully circumvented this masking effect, allowing the phage-specific additive signals to emerge above the control background.

#### 3.2.2 Phage-specific modulation of adaptive cytokines

Specific immunomodulatory effects were identified for T-cell-associated cytokines, revealing differential immunogenic potentials between the staphylococcal and coliphage preparations. Stimulation with the high dose of A5/80 resulted in a significant upregulation of IFN-γ (median: 33.96 pg/mL) compared to the matched control (17.09 pg/mL; *p*_adj_ = 0.0469) as well as T4 at the same dose (16.09 pg/mL; *p*_adj_ = 0.0469). An increase was also observed for IL-2 secretion in response to A5/80 (median: 8.25 pg/mL) relative to the LPS benchmark (3.79 pg/mL; *p*_exact_ = 0.0156, *p*_adj_ = 0.0469) and to the T4 preparation (3.49 pg/mL; *p_exact_* = 0.0156, *p_adj_* = 0.0469). This indicates that this phage may exert an immunostimulatory effect on the Th1 axis, whereas T4 is much weaker. The same trend was observed for IL-17A, where A5/80 at the high dose elicited a more pronounced response than T4 (5.06 pg/mL vs. 3.23 pg/mL; *p_exact_* = 0.0156, *p*_adj_ = 0.0938), whereas at the low dose it acted slightly stronger than control (1.39 pg/mL vs. 0.85 pg/mL; *p_exact_* = 0.0313, *p*_adj_ = 0.0938). This divergence in immunogenicity between those phage strains mirrors recent findings by Démoulins et al. (47). Using a bovine *ex vivo* model, they demonstrated that immune responses to bacteriophages are highly strain-dependent, with the coliphage T1 inducing profound immune activation, while the staphylococcal phage K remained immunologically silent. Interestingly, direct comparison of the high T4 dose to the control for IFN-γ secretion could even suggest a weak opposite effect. While the control cells secreted the cytokine at a median level of 17.09 pg/mL, the phage addition tended to reduce this response to 16.09 pg/mL, despite the equivalent background load (*p*_exact_ = 0.0313, *p*_adj_ = 0.0625). This aligns with earlier observations that T4 phage inhibits LPS-induced cellular activation (40), a phenomenon subsequently linked to T4 tail adhesins (gp12) physically binding LPS and counteracting endotoxin-induced inflammation *in vivo* (41).

#### 3.2.3 Regulatory cytokine profiles

Analysis of the anti-inflammatory IL-10 revealed a functional dichotomy between the two phage strains. For A5/80, the secretion profile was virtually indistinguishable from the background. The median IL-10 concentration (125.38 pg/mL) induced by the high phage dose was nearly identical to that of the control (125.08 pg/mL). This indicates that, unlike the synergistic boosting observed for Th1 cytokines, the release of IL-10 in A5/80-stimulated cultures is driven predominantly by the background stimuli rather than a specific viral mechanism. Conversely, the T4 phage exhibited an inhibitory profile. Despite the equivalent background load, stimulation with T4 at high titer resulted in a median IL-10 secretion of 50.36 pg/mL, exhibiting a distinct downward trend compared to the control (125.08 pg/mL; *p*_exact_ = 0.0273, *p*_adj_ = 0.0820). This suppressive profile of the coliphage was further highlighted when directly compared to the staphylococcal preparation; A5/80 elicited a notably higher IL-10 response than T4 at the high dose (*p*_exact_ = 0.0273, *p*_adj_ = 0.0820). Although statistical significance was attenuated following FDR correction, the uncrossed HL confidence intervals in both comparisons suggest phage-specific dampening of this regulatory cytokine by T4. This finding is biologically consistent with the suppression of IFN-γ observed in the same cultures; the lack of a robust pro-inflammatory trigger likely obviates the need for a compensatory anti-inflammatory feedback loop (48). Other cytokines, including IL-4 and IL-5, exhibited baseline secretion across all tested conditions. Although A5/80 high-dose condition exhibited a nominal upward trend in median IL-21 secretion compared to the matched control (median: 25.29 pg/mL vs. 19.37 pg/ml; *p*_exact_ = 0.0312, *p*_adj_ = 0.1406), the absolute concentrations remained low. Given that all median values for this cytokine fell below the assay-specific LLQ, these minor fluctuations appear to be of limited biological relevance, particularly when contrasted with the robust secretion magnitude elicited by a replicating viral pathogen (see Section 3.9). We also observed nominally higher IL-13 secretion induced by the staphylococcal phage than by the *E. coli* phage at the high dose (33.06 vs. 23.15 pg/mL; *p*_exact_ = 0.0313, *p*_adj_ = 0.1875). Despite this modest phage-dependent difference, neither phage induced a robust absolute signal above the endotoxin background.

### 3.3 Cytokine secretion profiles of monocytes

Monocytes exhibited a pronounced activation profile compared to the total PBMC population. While the overall magnitude of cytokine secretion was substantial across all treated cell groups, the monocyte-enriched fraction revealed a specific sensitivity especially to the T4 phage, which frequently elicited responses exceeding the already high magnitude of cytokine release induced by the matched controls (**Figure 2**).

**Figure 2:**
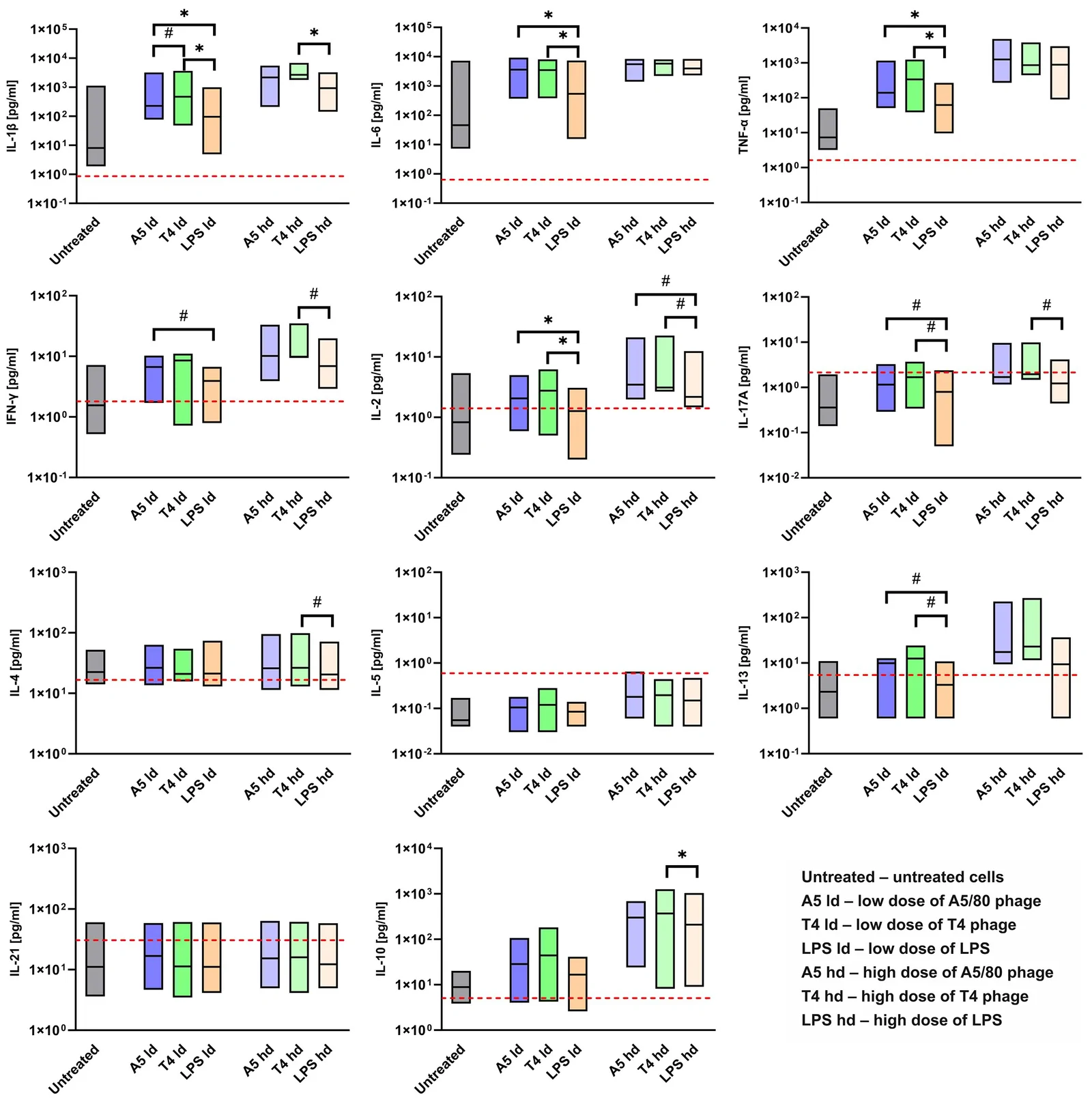
Cytokine secretion profiles of isolated monocytes. The internal horizontal line indicates the median value, while the upper and lower boundaries of the bar represent the absolute maximum and minimum values, respectively. Brackets mark statistical significance: ^#^denotes a nominal trend (*p_exact_* < 0.05); *indicates significant difference (*p_adj_* < 0.05) after BH-FDR adjustment. The dashed red line indicates the assay-specific lower limit of quantification.

#### 3.3.1 Detection of lymphoid-associated cytokines

Although the isolated cells were predominantly monocytes, we observed the secretion of cytokines canonically restricted to lymphoid cells, namely IFN-γ, IL-2, IL-13, and IL-17A. For IFN-γ, both stimulation with high-dose T4 and low-dose A5/80 phages resulted in an upward shift in median cytokine secretion compared to their matched controls (9.86 pg/mL vs. 6.89 pg/mL, and 7.08 pg/mL vs. 3.915 pg/mL, respectively; *p*_exact_ = 0.0313, *p*_adj_ = 0.0938 for both). While studies suggest that human monocytes can produce IFN-γ upon specific stimulation (49), we cannot exclude the possibility that this signal partially originates from the paracrine activation of residual lymphocytes by monocyte-derived cytokines (e.g., IL-12/IL-18) (50). A similar pattern was noted for IL-2, IL-13, and IL-17A, where phage-treated samples consistently exceeded the controls. Given their lymphoid restriction and near-baseline levels, this signal likely reflects this same bystander rather than an atypical secretion by monocytes.

#### 3.3.2 Pro-inflammatory innate responses

In contrast to the general PBMC results, where innate cytokines mirrored the matched controls, isolated monocytes demonstrated a specific responsiveness to phages. For IL-1β, stimulation with the high dose of T4 resulted in a median secretion of 2683 pg/mL, significantly surpassing the 926 pg/mL elicited by the matched high-dose control (*p*_exact_ = 0.0156, *p*_adj_ = 0.0313). Notably, the high dose of A5/80 also drove an elevation in median secretion (2169 pg/mL), although the difference remained statistically non-significant. Direct comparison between the two high-dose phage preparations did not reveal a difference in IL-1β induction. At lower titers, both A5/80 and T4 significantly increased IL-1β secretion to median levels of 229 pg/mL and 470 pg/mL, respectively, compared to the low-dose control of 96 pg/mL (*p*_exact_ = 0.0156, *p*_adj_ = 0.0313 for both). Interestingly, a direct comparison of both phage preparations at their lower doses trended toward a stronger induction of this cytokine by T4 (median 470.31 pg/mL vs. 228.96 pg/mL; *p*_exact_ = 0.0469, *p*_adj_ = 0.0703), further highlighting the specific reactivity of monocytes to the coliphage preparation.

For TNF-α, a distinct pattern of responsiveness was evident, particularly at lower phage titers, where the specific phage-mediated signal clearly emerged above the control. Both A5/80 and T4 at low doses induced median secretion levels of 138.99 pg/mL and 336.10 pg/mL, respectively, which significantly surpassed the corresponding low-dose matched control of 62.34 pg/mL (*p*_exact_ = 0.0156, *p*_adj_ = 0.0469 for both). Conversely, at high doses, the responses converged probably with the physiological saturation points. Median secretion levels showed no statistically significant amplification for either A5/80 (1256.21 pg/mL) or T4 (859.15 pg/mL) compared to 884.42 pg/mL elicited by the matched high-dose benchmark. The direct comparison between both phage preparations at the high- and low-doses also remained non-significant.

Regarding IL-6 secretion, a statistically significant increase was observed exclusively at low phage titers. The T4 and A5/80 preparations yielded median levels of approximately 3500–3600 pg/mL, significantly higher than the 537.74 pg/mL observed for the corresponding control (*p*_exact_ = 0.0156, *p*_adj_ = 0.0469 for both). At high doses, however, absolute cytokine concentrations reached extreme levels, with individual donor responses frequently exceeding 6000–8000 pg/mL. This ceiling effect, reflecting physiological and/or assay saturation, precluded any statistical differentiation from the already maximized control.

#### 3.3.3 Regulatory and Th2 polarization

A distinct immunomodulatory signature was observed for the T4 phage regarding regulatory and type 2 cytokines. Unlike in the total PBMC population, where IL-10 modulation was variable, isolated monocytes stimulated with the high dose of T4 showed a significant upregulation of this anti-inflammatory cytokine. Specifically, it induced a median IL-10 secretion of 200.14 pg/mL, significantly surpassing the 68.68 pg/mL elicited by the corresponding control (*p*_exact_ = 0.0078, *p*_adj_ = 0.0469). Importantly, this tolerogenic profile was further supported by trends within the monocyte population.

A nominal elevation of IL-4 was also observed for the high dose of T4 phage compared to its control (median 26.4 pg/mL vs. 20.470 pg/mL; *p*_exact_ = 0.0156, *p*_adj_ = 0.0938). However, classical monocytes are not a recognized cellular source of IL-4; rather, it canonically acts upon them as an exogenous polarizing stimulus (51). Given the near-baseline secretion levels and the lack of a distinct phage-strain specificity, this signal probably reflects the bystander activation of trace residual cells (e.g., Th2 lymphocytes) co-purified during negative selection. While minor, this paracrine presence of IL-4 may exert a localized regulatory effect on the monocyte population, a phenomenon that is further explored in the Discussion.

### 3.4 Cytokine secretion profiles of T lymphocytes

To capture the aggregate response of the T-lymphocyte compartment, we first evaluated the total CD3^+^ pool (n= 3). In these cultures, cytokine secretion remained noticeably lower than in whole PBMCs, likely reflecting the dependency of robust *ex vivo* T-cell activation on co-stimulation from accessory antigen-presenting cells (**Figure 3**). Despite this generally muted baseline, stimulation with the high-titer staphylococcal phage triggered a discernible upward trend in adaptive cytokines (IFN-γ, IL-17A) relative to the control (a profile we subsequently explored in detail within the isolated CD4^+^ subpopulation). In contrast, the T4 phage consistently elicited negligible responses comparable to the controls, consistent with the non-inflammatory profile of this coliphage observed across all lymphoid fractions. Similar to other purified subpopulations, sporadic elevations of innate markers (IL-6, TNF-α) were observed but remained within the range of the benchmark. Furthermore, type 2 cytokines (IL-4, IL-5, IL-13) remained consistently low or undetectable across all donors and experimental conditions, suggesting that neither phage induces a Th2-skewed response. The observed heterogeneity, particularly the lack of response in some donors to standard stimuli, likely reflects variations in the residual fraction of accessory antigen-presenting cells required for robust T-cell activation *ex vivo*.

**Figure 3:**
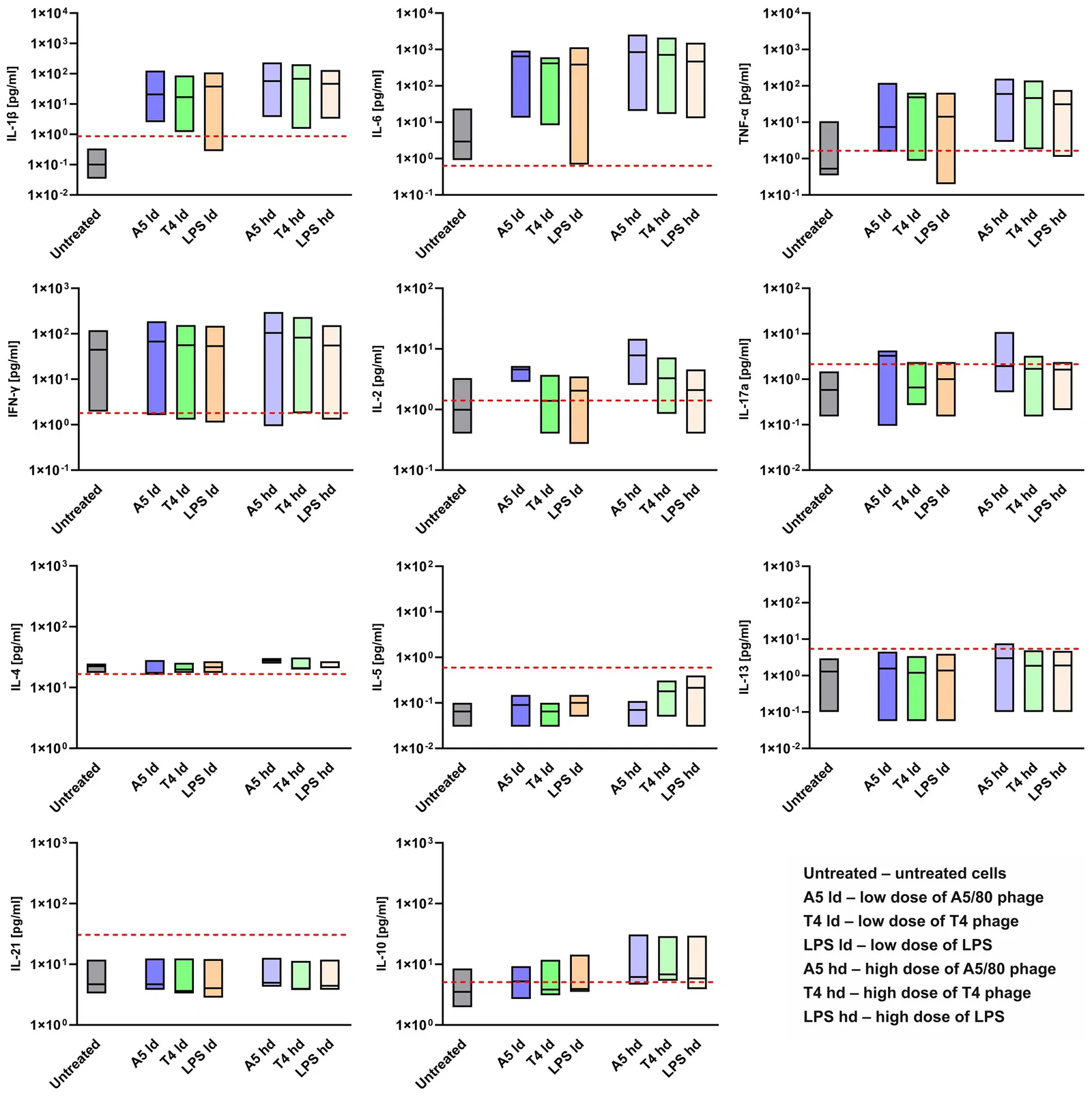
Cytokine secretion profiles of T-lymphocytes. The internal horizontal line indicates the median value, while the upper and lower boundaries of the bar represent the absolute maximum and minimum values, respectively. The dashed red line indicates the assay-specific lower limit of quantification.

### 3.5 Cytokine secretion profiles of T helper cells

To characterize the adaptive arm of the immune response, cytokine secretion was analyzed in untouched CD4^+^ cells from 7 healthy donors (**Figure 4**). Consistent with observations in the total PBMC population, the data exhibited high inter-donor variability. One donor displayed a “super-responder” phenotype with cytokine levels orders of magnitude higher (e.g., IFN-γ > 6000 pg/mL) than the cohort median. However, as the directional trends in this donor were consistent with the rest of the group, the subject was retained in the analysis to reflect natural biological heterogeneity. An extreme inter-individual variance spanning multiple orders of magnitude specifically for IFN-γ production is a well-documented feature even among healthy cohorts (52).

**Figure 4:**
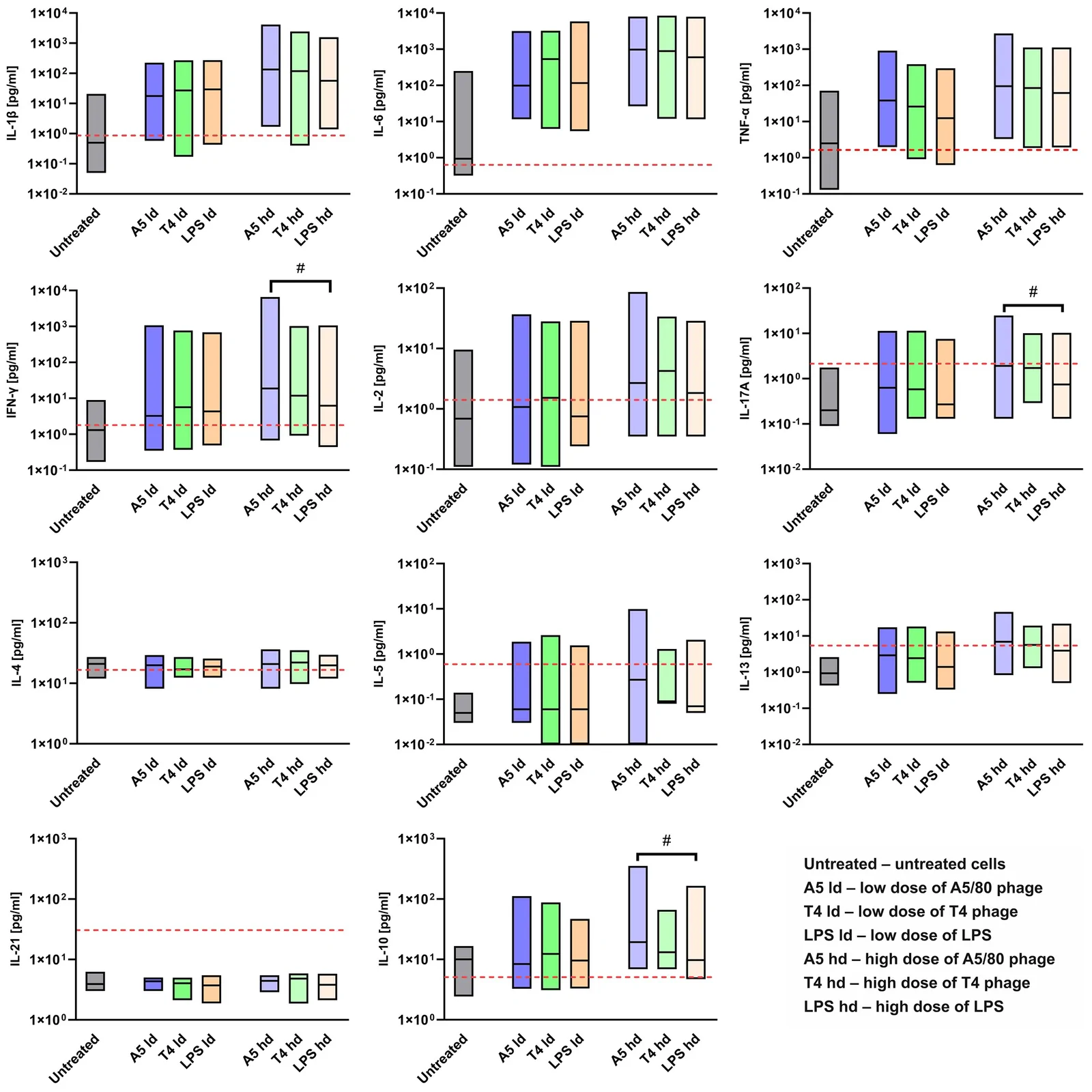
Cytokine secretion profiles of isolated T helper lymphocytes. The internal horizontal line indicates the median value, while the upper and lower boundaries of the bar represent the absolute maximum and minimum values, respectively. Brackets mark statistical significance: ^#^denotes a nominal trend (*p_exact_* < 0.05); *indicates significant difference (*p_adj_* < 0.05) after BH-FDR adjustment. The dashed red line marks the mean lower limit of quantification.

#### 3.5.1 Th1-biased activation profile

Exposure to the high-titer A5/80 phage preparation induced a nominal increase in Th1-associated cytokine secretion. The median level of IFN-γ showed an increase compared to the reference control (median: 18.76 pg/mL vs. 6.21 pg/mL; *p*_exact_ = 0.0469; *p*_adj_ = 0.2812). This activation profile mirrors the upregulation of immune-related genes by this specific phage strain, as previously reported in epithelial cell models (10). Conversely, secretion of the Th17-associated cytokine IL-17A remained at trace levels across all conditions (medians high-dose< 2.0 pg/mL), indicating no relevant activation of this specific pathway. Given the presence of minor residual non-target cells in the isolated fractions (up to 8%), we cannot exclude that this Th1-biased profile represents a rapid paracrine signaling loop initiated by trace APCs rather than strictly autonomous T-cell sensing.

#### 3.5.2 Innate and pro-inflammatory signals

In contrast to the specific adaptive cytokines, the secretion of general pro-inflammatory mediators (IL-6, TNF-α, IL-1β) showed a uniform, non-specific pattern. A universal induction of IL-6 was observed across all stimuli, with both phages and controls eliciting comparable, high-level secretion. Similarly, while non-significant elevations of TNF-α were noted for both the high-dose A5/80 and T4 phages, these responses did not statistically differ from the benchmark.

#### 3.5.3 Regulatory and Th2 cytokines

A5/80 high-dose stimulation elicited a median IL-10 secretion of 19.28 pg/mL, displaying a nominal increase compared to the 9.74 pg/mL observed in the control cells (*p*_exact_ = 0.0469; *p*_adj_ = 0.2813). This functionally mirrors the specific induction of anti-inflammatory pathways (such as *TLR10* expression) noted for this staphylococcal phage in other *in vitro* studies (10). Together, these findings highlight a mixed pro-inflammatory and self-regulating potential unique to the staphylococcal phage preparation. Regarding Th2-associated cytokines, responses were muted or constitutive. IL-5 secretion remained minimal across all conditions. Interestingly, IL-4 and IL-21 displayed a distinct profile characterized by high constitutive secretion, even in unstimulated cells. Neither phage stimulation nor the controls significantly modulated these high baseline levels, suggesting that these pathways are not primary targets of phage-mediated signaling in resting T-helper cells.

### 3.6 Cytokine secretion profiles of cytotoxic T lymphocytes

Given that the activation of cytotoxic T lymphocytes canonically requires intracellular antigen processing and presentation via HLA class I molecules, we expected that extracellular bacteriophages would elicit minimal direct responses in this fraction. Our *ex vivo* preliminary data support this hypothesis (n = 3, **Figure 5**).

**Figure 5:**
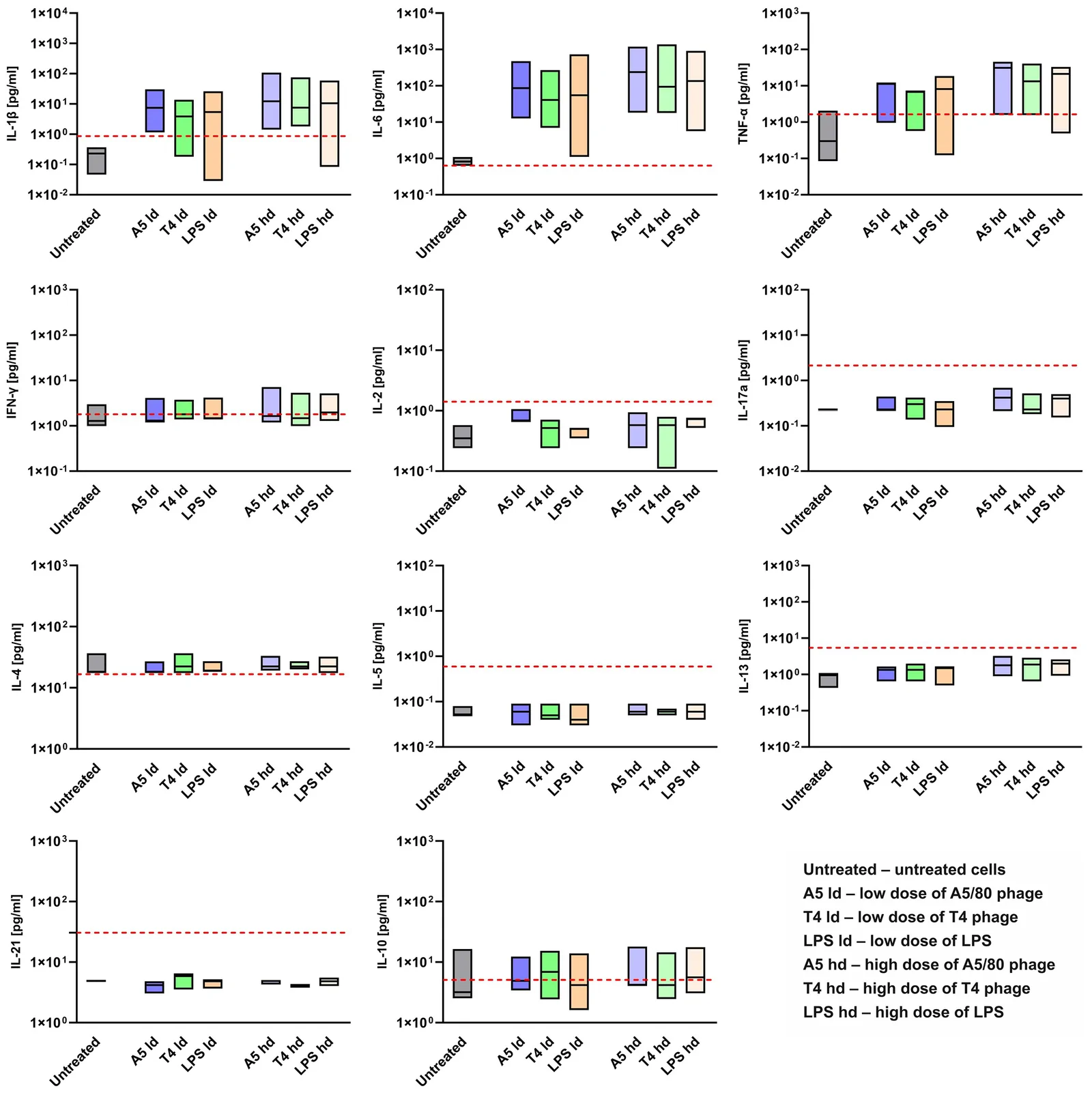
Cytokine secretion profiles of cytotoxic lymphocytes. The internal horizontal line of the bar indicates the median value, while the upper and lower boundaries represent the absolute maximum and minimum values, respectively. The dashed red line indicates the assay-specific lower limit of quantification.

Secretion of effector Th-1 cytokines was negligible. IFN-γ levels remained below 7 pg/mL, lower than typical polyfunctional antiviral responses (53) or the responses observed in our total T lymphocyte fraction (> 300 pg/mL). While trace levels of innate markers (IL-6, TNF-α, IL-1β) were detected in certain samples, this profile aligns with the bystander activation of residual accessory myeloid cells rather than intrinsic Tc signaling. Resting human cytotoxic T cells lack robust surface expression of innate pattern recognition receptors, such as TLR2 and TLR4, and require prior activation for direct responsiveness (54, 55). This physiological restriction is corroborated by the negligible cytokine secretion elicited by the LPS control in these cultures. Furthermore, no detectable secretion of type 2 cytokines (IL-4, IL-5, IL-13) was observed. Together, these data are consistent with the lack of a direct cytokine response by Tc lymphocytes to extracellular phage challenge.

### 3.7 Cytokine secretion profiles of B lymphocytes

In the initial screening of B cell-enriched cultures (n = 3), cytokine secretion profiles did not exhibit a pronounced trend of phage-specific activation. Specifically, the levels of innate pro-inflammatory mediators, including IL-6, TNF-α, and IL-1β, showed no upregulation in phage-treated cultures (high-dose A5/80 and T4) and mirrored their corresponding benchmarks (**Figure 6**). This secretion pattern suggests that the purified B-cell fraction does not mount a specific response to the virions of the T4 or A5/80 phages. The observed cytokine release is likely attributed to the activity of highly responsive residual accessory cells (such as trace monocytes) that persist within the non-B-cell fraction. Based on our quality control, this non-target fraction constituted approximately 9% of the isolated populations.

**Figure 6:**
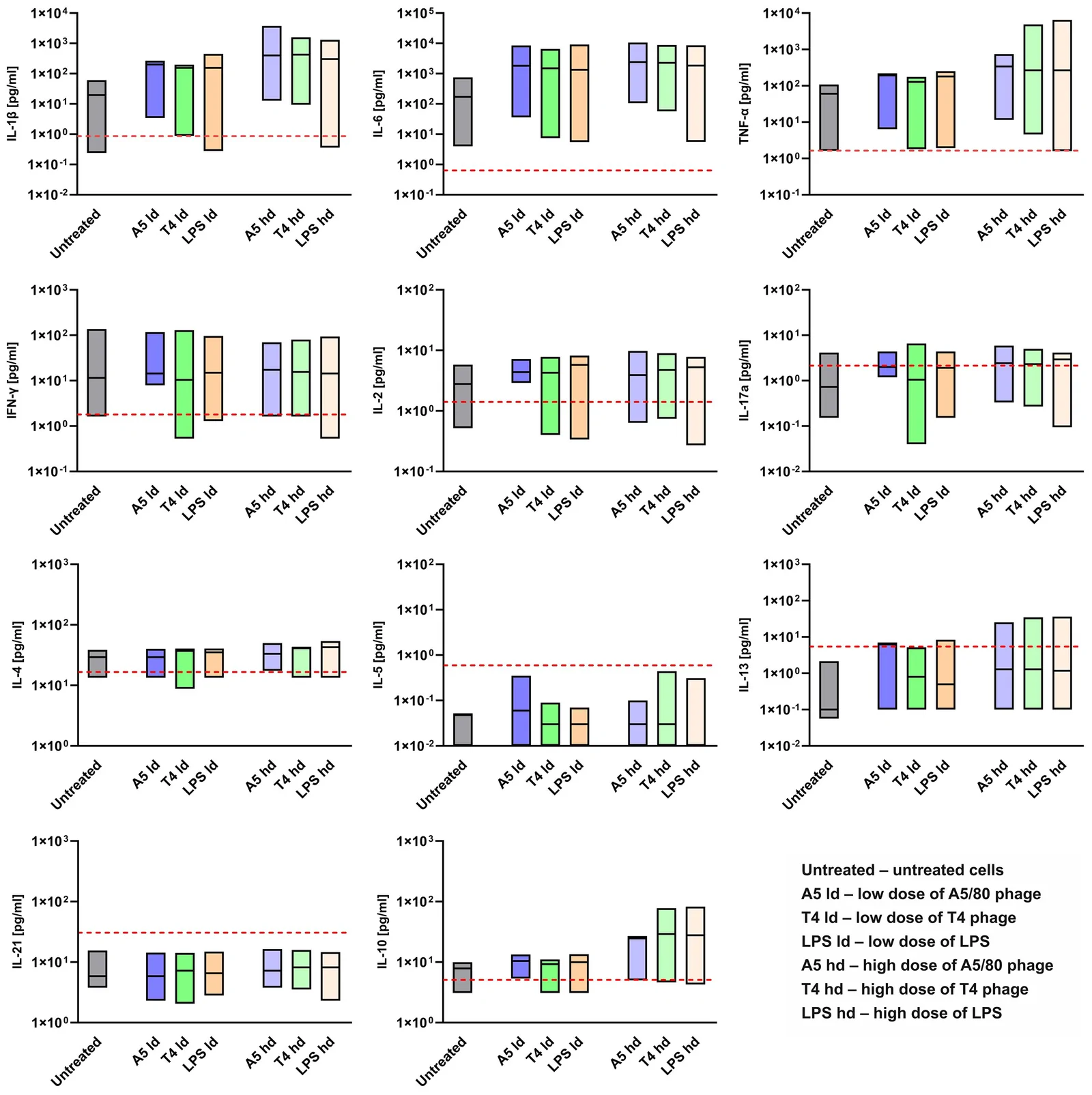
Cytokine secretion profiles of B lymphocytes. The internal horizontal line indicates the median value, while the upper and lower boundaries of the bar represent the absolute maximum and minimum values, respectively. The dashed red line indicates the assay-specific lower limit of quantification.

Biologically, resting human B cells express low levels of innate pattern recognition receptors (such as TLR4 and TLR2) (56), which may limit their direct responsiveness through these pathways under the experimental conditions used here. In contrast, monocytes are highly sensitive and can produce nanogram quantities of cytokines even when present in low numbers. Consequently, the marked secretion of TNF-α and IL-6 observed in some donors may primarily reflect the activation of a small number of residual monocytes rather than direct B-cell activation.

This lack of response was further supported by the analysis of adaptive immunity markers. Cytokines associated with T-cell help or Th2 polarization, specifically IL-2, IL-4, IL-5, and IL-21, remained at baseline levels or near the lower limit of quantification across all experimental conditions. The absence of IL-21 is particularly relevant in this context. As IL-21 is produced by follicular helper T cells to drive B-cell differentiation and proliferation, its absence confirms that the pro-inflammatory signals observed in the cultures occurred independently of T-cell contamination or exogenous help.

### 3.8 Cytokine secretion profiles of NK cells

Natural killer cells typically require a dual activation signal: direct receptor engagement coupled with cytokine support (primarily IL-12 and IL-18) provided by accessory myeloid cells (57). In our isolation model using negative selection, where such accessory signals were deliberately minimized, we assessed the capacity of bacteriophages to act as stand-alone activators (n = 3-4, **Figure 7**).

**Figure 7:**
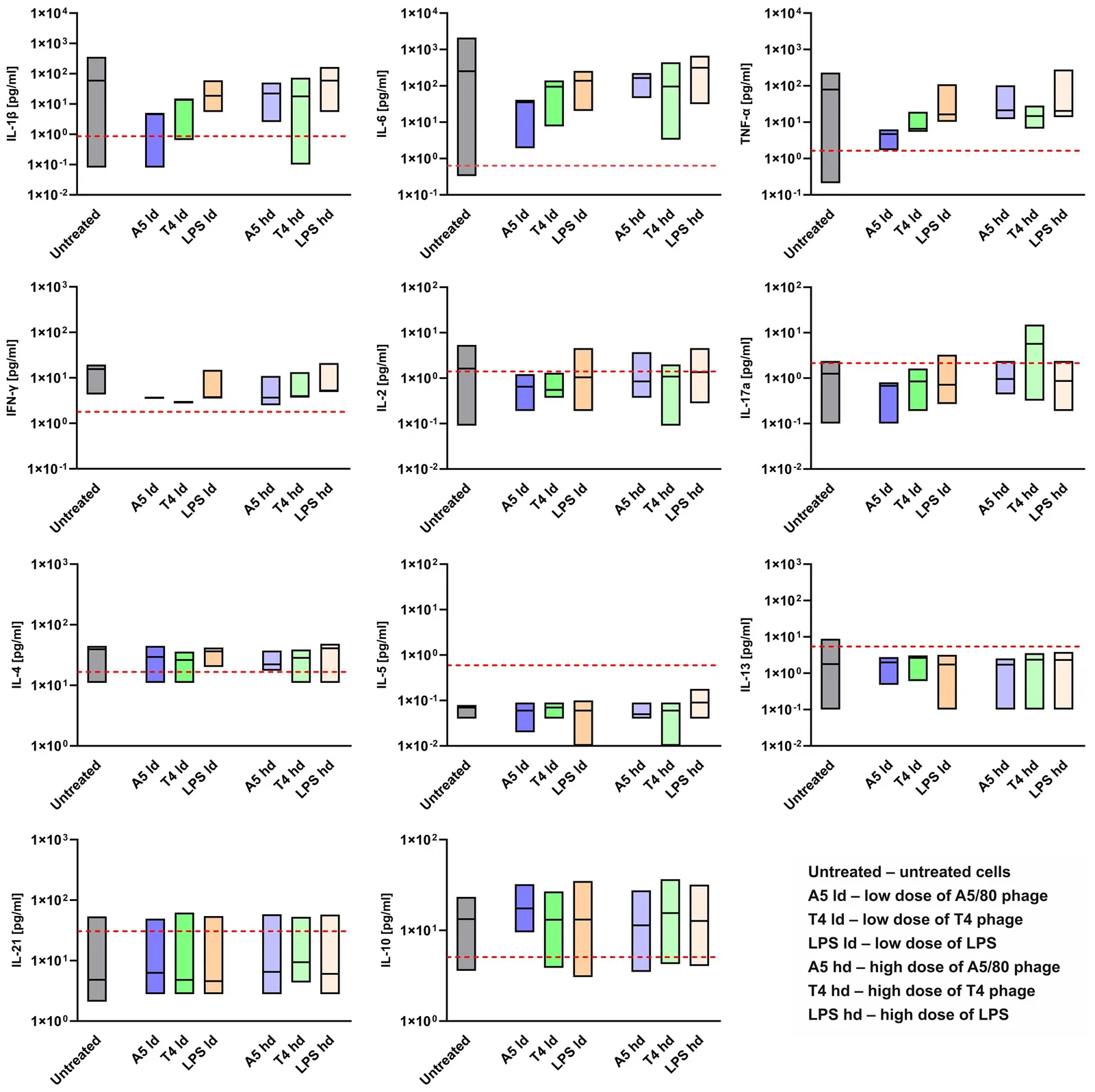
Cytokine secretion profiles of NK cells. The internal horizontal line indicates the median value, while the upper and lower boundaries of the bar represent the absolute maximum and minimum values, respectively. The dashed red line indicates the assay-specific lower limit of quantification.

The results reveal a lack of direct phage-driven activation across all conditions. Notably, regarding the hallmark NK cytokine IFN-γ, secretion levels were negligible, remaining at a median of < 20 pg/mL even in the presence of the robust high-dose positive control. This stands in contrast to the distinct IFN-γ release typically observed in PBMC cultures (**Figure 1**), indicating that the removal of CD14^+^ accessory cells disrupted the indirect activation loop. Neither the T4 nor the A5/80 phages induced IFN-γ secretion above this low baseline, suggesting that these virions do not possess intrinsic motifs capable of triggering NK effector functions in the absence of co-stimulation. Similar to other lymphoid fractions, the innate cytokine background (IL-6, TNF-α) was driven by the donor-specific sensitivity of residual accessory cells, while the IFN-γ remained unresponsive to direct phage stimulation. The addition of phages to these cultures did not exacerbate cytokine release; trajectories generally stabilized or trended downward compared to the untreated cells. Finally, consistent with the non-inflammatory profile observed in other lymphoid fractions, no secretion of Th2-associated cytokines (IL-4, IL-5) or IL-21 was detected. Given this lack of direct cytokine induction, future studies should evaluate the immunomodulatory impact of phages by assessing primary NK effector functions, specifically CD107a degranulation (58) and direct cytotoxicity against target cells – ideally within complex co-culture systems that preserve physiological immune cross-talk.

### 3.9 Comparison of PBMC activation by bacteriophages and adenovirus

To contextualize the absolute magnitude of phage-induced immune activation, a descriptive pilot experiment was conducted using PBMCs from a single donor, with AdV-5 serving as a potent viral reference stimulus. This comparison should be interpreted in light of the mechanistic differences between these viruses. Whereas AdV-5 is a human pathogen capable of actively entering and replicating within permissive mammalian cells, thereby triggering robust danger signals (e.g., via cytosolic DNA sensors) (59), the bacteriophages used here act primarily as extracellular pathogen-associated molecular patterns (PAMPs) or inert particles in the context of mammalian cells (60). Therefore, AdV-5 was used not to imply biological equivalence but strictly as a benchmark for the magnitude of the antiviral cytokine response in our *ex vivo* PBMC model. The selection of AdV-5 as a reference is supported by both structural homologies and evolutionary connections. Complex bacteriophages and human adenoviruses share architectural principles: both are non-enveloped, double-stranded DNA viruses that utilize conserved structural solutions to package and deliver their genomes (61, 62). Furthermore, these two viruses exhibit similarities in their interactions with mammalian host cell surfaces. Specific phage structural proteins, such as the T4 capsid protein gp24, harbor a Lys-Gly-Asp (KGD) amino acid motif known to bind to integrins (63). Adenoviruses also use integrins, including αvβ3 and αv5β5, as co-receptors for cellular internalization. This overlap provides an additional rationale for using AdV-5 as a viral reference stimulus (20, 42).

AdV-5 was applied at nominal input doses selected to correspond approximately to the high and low titers of the bacteriophage preparations. Specifically, cells were challenged with AdV-5 at a high titer of 3.5 × 10^8^ PFU/mL and a low titer of 3.5 × 10^7^ PFU/mL (estimated as described in the Materials and Methods section). This does not imply perfect biological equivalence in terms of cell entry or innate sensing dynamics. Although the stock AdV-5 preparation was verified to be free of detectable endotoxin contamination (< 0.01 EU/mL), it was produced by repeated freeze-thawing of A549 cells, and no corresponding mock-infected control preparation was included. Therefore, the preparation may have contained mammalian host-derived proteins, nucleic acids, and other components. Consequently, the pronounced cellular activation observed likely represents a combined response to the viral challenge and these damage-associated molecular patterns.

As shown in **Figure 8**, which highlights the key antiviral cytokines IFN-γ and IL-21, the cytokine response to phage preparations was markedly lower than that observed with the AdV-5 preparation. Stimulation with the low-titer AdV-5 induced IFN-γ secretion of approximately 220 pg/mL and IL-21 secretion of ~20 pg/mL. The high-titer AdV-5 was even more potent, inducing approximately 450 pg/mL of IFN-γ and ~40 pg/mL of IL-21. These levels are substantially higher (7- to 17-fold for the low-titer and 13- to 35-fold for the high-titer AdV-5) than those observed with any phage preparation alone. Because the phage preparations contained residual endotoxin that may have provided an additive pro-inflammatory signal, the intrinsic immunostimulatory activity of the phage virions may therefore be lower than suggested by the aggregate response. This pilot comparison serves strictly to contextualize the magnitude of the overall response measured, demonstrating that while specific phage preparations (particularly A5/80) did induce statistically significant increases in cytokines like IFN-γ, the absolute scale of this secretion is minimal and distinct from the potent inflammatory cascade triggered by an established viral pathogen.

**Figure 8:**
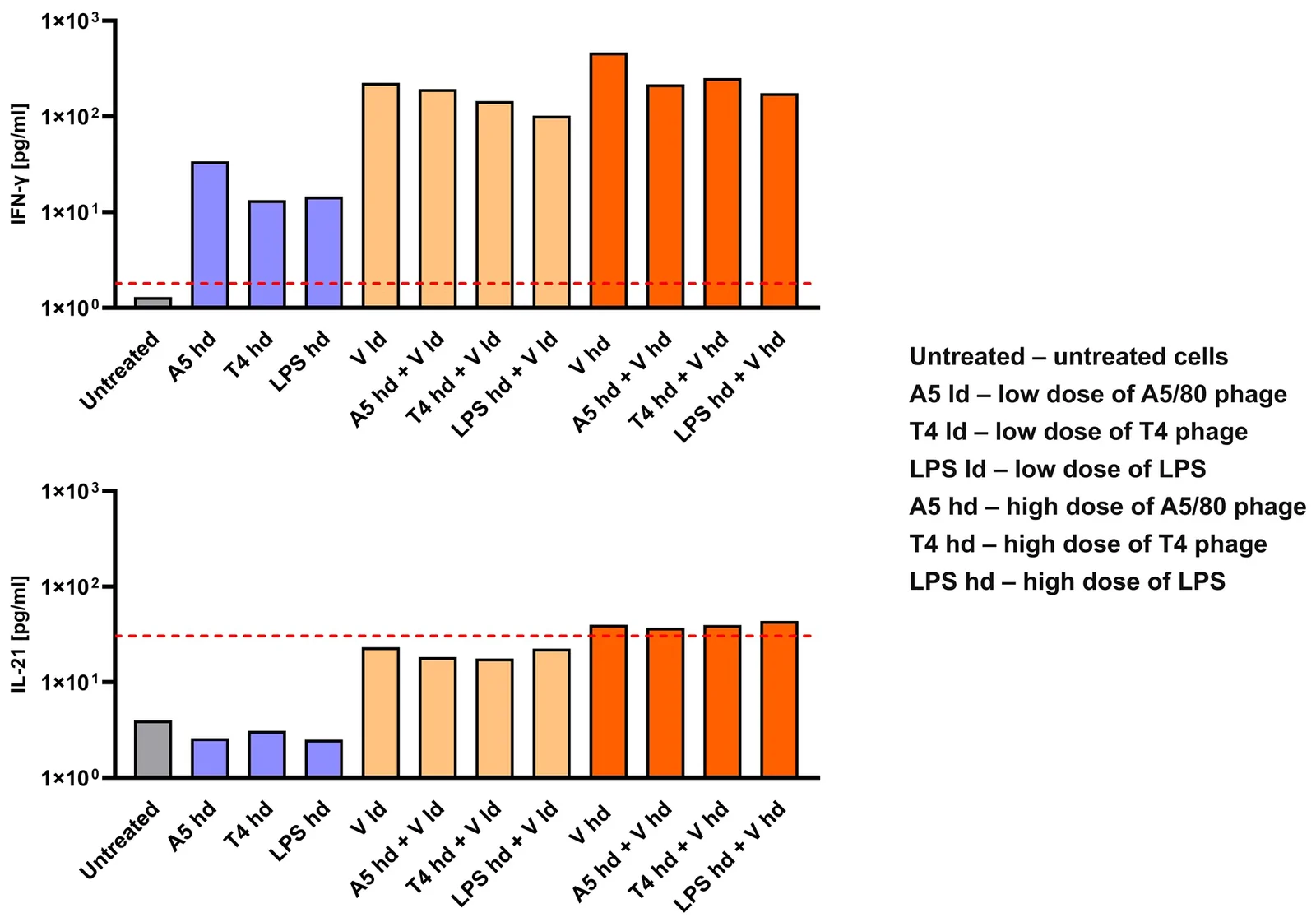
Contextualizing the magnitude of phage-induced immune activation. Supernatant concentrations of IFN-γ and IL-21 secreted by PBMCs from a single healthy donor in response to high-dose of A5/80 and T4 phages, alongside the matched high dose of LPS control, are shown relative to the responses elicited by a high- and low dose AdV-5 challenge.

Interestingly, the co-incubation setup revealed that the presence of bacteriophage preparations did not exacerbate the AdV-5-induced inflammatory cytokine response. Instead, a modulatory trend was observed; for example, IFN-γ and IL-13 levels induced by AdV-5 were visibly dampened when co-incubated with A5/80 or T4 phage preparations. However, because a similar dampening effect was observed in the matched LPS controls, this regulatory trend is likely driven by the residual bacterial components rather than a specific interaction with the phage virions. The expanded cytokine panel from this challenge experiment, showing the differences in response magnitude and the modulatory trends for the other cytokines, is provided in **Supplementary Figure S4**.

## 4 Discussion

Our findings corroborate the “phage repurposing” concept, which extends the role of bacteriophages beyond their canonical antibacterial action (2). Rather than being immunologically inert, phages possess the capacity to directly interact with eukaryotic cells and act as modulators of the host immune system. This non-antibacterial activity manifests functionally through the modulation of specific immune cell networks (2, 64). In this context, our data indicate that the T4 and A5/80 phage preparations trigger and shape distinct cytokine responses in human immune cells. This aligns with the growing body of evidence characterizing phages as complex biological nanoparticles carrying specific structural motifs recognized by mammalian pattern recognition receptors (65). Their immune recognition is not a generic effect but is linked to immunogenic structural components, particularly T4 capsid proteins like Hoc and gp23* (41), and genomic DNA (66). While phage DNA has generally been shown to be sensed by intracellular pattern recognition receptors (PRRs), such as the endosomal TLR9 and the cytosolic cGAS-STING pathway (67), data indicate that specific phages, including T4, can be internalized without triggering these inflammatory DNA-sensing cascades (66).

To accurately capture this immunological complexity, we utilized an *ex vivo* primary human cell model, providing a more physiologically relevant assessment than immortalized cell lines, which are documented to exhibit altered responsiveness (68). Within this *ex vivo* system we observed that the tested phage preparations elicit distinct, cell fraction-dependent cytokine signatures. However, the absence of physiological tissue barriers, endothelial layers, and systemic immune cross-talk means that the observed basal responses to purified virions may differ from those within a living organism. Furthermore, clinical phage therapy typically involves bacterial lysis and local phage bursts, triggering locally inflammatory cascades (21, 69). This dynamic, which paradoxically may finally lead to therapeutic success, represents a complex immunomodulatory environment that cannot be captured in a pathogen-free system. Regarding temporal resolution, this study relies on a single 24-hour endpoint, providing a static snapshot of a highly dynamic immune response. Kinetic analyses demonstrate that cytokine secretion is strictly time-dependent; early transient pro-inflammatory signals (such as TNF-α) typically peak within the first 9 hours post-stimulation, whereas regulatory feedback loops driven by IL-10 mature closer to 24 h (70). Consequently, our 24-hour measurement may underestimate immediate, early-phase pro-inflammatory bursts, but it is optimally positioned to capture sustained pro-inflammatory signaling and the subsequent regulatory responses. Thus, the observed cytokine milieu reflects a more mature, potentially self-regulating stage of the immune reaction to bacteriophages.

### 4.1 Cellular hierarchy and the two-tiered model of phage recognition

An important aspect of this study is the high-resolution analysis of cytokine secretion at the level of individual immune cell subpopulations, which moves beyond the limitations of bulk PBMC assays. Our data suggest a distinct cellular hierarchy in the response to bacteriophages, with monocytes appearing to act as primary cytokine producers in this model. These primary responders drove a robust secretion of classical innate mediators (such as IL-1β, IL-6, and IL-10), while acting as the focal point for initiating broader downstream cytokine cascades. This finding lends support to the hypothesis that only specific immune subsets have specialized as primary sentinels for bacteriophages. As professional phagocytes and antigen-presenting cells, monocytes are uniquely equipped with a vast repertoire of PRRs, such as TLRs capable of recognizing phage nucleic acids, positioning them as the innate first responders. Similar immunomodulatory rewiring has been recently described for other exogenous antimicrobial agents; for instance, the antimicrobial peptide BSN-37 promotes macrophage activation and the secretion of classical innate mediators (such as IL-1β, IL-6, and TNF-α) by remodeling nuclear non-coding RNA networks, specifically through the circBptf-Rbm14 regulatory axis (71). This highlights that the internalization of foreign particles by professional phagocytes can trigger complex intracellular cascades involving RNA-protein interactions, providing a plausible mechanistic framework for how specific bacteriophages might fine-tune monocyte activation and subsequent cytokine signatures.

In contrast to the broad, primary sensing role of monocytes, the contribution of T-helper lymphocytes, which also produced IFN-γ and IL-10, reflects a secondary, more tailored phase of the immune response (72). Canonically, T-helper cell activation is dependent on antigen presentation by professional APCs, such as monocytes (73). Their cytokine production in our *ex vivo* system is likely a downstream consequence of signals initiated and orchestrated by monocytes. This two-tiered model is supported by single-cell studies of classical innate immune stimuli. Therefore, we functionally attribute the cytokine secretion observed in the enriched T helper cultures primarily to an indirect, paracrine activation driven by the minor fraction of residual accessory cells inherent to the *ex vivo* isolation process. This points toward a potential two-tiered immune reaction: an immediate innate response by these primary sentinels, which in turn orchestrates and shapes the subsequent adaptive response by T lymphocytes. However, while our dataset relies solely on secreted cytokine concentrations and does not directly evaluate receptor activation or HLA-DR-dependent contact, the observed patterns allow us to propose this biologically plausible, yet untested, schematic hypothesis.

Our data indicate a notable and context-dependent immunomodulatory effect of the T4 bacteriophage. In monocyte-enriched cultures, the phage preparation significantly increased the secretion of the anti-inflammatory cytokine IL-10, which suggests a direct action on innate antigen-presenting cells, consistent with literature indicating that pathogens can trigger IL-10 production in macrophages via TLR and p38 MAPK pathways (74). Concurrently, a minor elevation of the Th2-polarizing cytokine IL-4 was detected, likely originating from paracrine bystander activation. Together, this initial response appears to be an immunosuppressive mechanism aimed at establishing a regulatory, tolerogenic microenvironment. From a clinical perspective, this specific induction of a tolerogenic baseline by phages like T4 aligns with hypotheses regarding their potential application as supportive immunomodulators in scenarios requiring immune dampening, such as post-transplant care or autoimmune conditions (44). In contrast, within the mixed PBMC environment, exposure to T4 phage resulted in a trend toward reduced of IL-10 secretion. This reversal underscores the multifaceted nature of IL-10, where the net outcome in a complex cellular environment, containing lymphocytes, is inverted. This phage-induced downregulation of IL-10 in PBMCs may represent a beneficial switch, overcoming the initial suppressive innate response to facilitate the rapid amplification of innate and adaptive immunity necessary for effective pathogen clearance (75).

This pattern of innate activation versus adaptive modulation was distinctly reflected in the secretion of IL-13. In monocyte-enriched cultures, stimulation with bacteriophages induced a discernible upward shift in IL-13 secretion. Notably, at lower viral doses, both T4 and A5/80 phages showed a trend toward increasing IL-13 levels. In contrast, IL-4 concentrations hovered near the lower limit of quantification across all conditions, indicating that the observed response is specifically driven by IL-13. This localized secretion raises the possibility of paracrine cross-talk within the culture. However, because specific MHC-restricted cross-talk and receptor activation were not directly evaluated in our assays, this remains an untested hypothesis.

The observed activation within the Th cell-enriched fraction, contrasted with the relative lack of response in B, NK, and Tc cell-enriched cultures, aligns with this schematic hypothesis. Given the 92–98% purity of our negative selection, the isolated subpopulations inherently contained 2–8% residual non-target cells, including trace APCs. It is plausible that this minor fraction of accessory cells is biologically sufficient to facilitate the observed Th-cell response through localized antigen presentation. However, further studies employing intracellular cytokine staining with lineage markers—as opposed to bulk secreted cytokine measurements—are required to definitively assign the cellular source of the cytokines and formally confirm the sequence of cellular cross-talk.

### 4.2 Niche-driven divergence in phage immune profiles

While our data demonstrate that the T4 and A5/80 preparations elicited distinct cytokine profiles relative to their respective endotoxin controls, direct statistical comparisons between the two preparations were additionally performed to comprehensively map the observed response ranges. However, these direct comparisons must be interpreted with caution. The differing propagation hosts (*E. coli* versus *S. aureus*) and varying bacterial backgrounds inherently confound the direct attribution of immunological differences solely to the phage virions. Thus, the observed divergences may reflect aggregate interactions with the complex phage preparations rather than purely virion-specific immune responses.

Within this context, we hypothesize that these contrasting profiles align with the distinct ecological niches of these phages and their bacterial hosts. The relatively subdued cytokine induction associated with the enteric coliphage T4 is consistent with an adaptive strategy to avoid excessive immune provocation within the heavily colonized, tolerogenic environment of the gut. Such conservative immunomodulation is supported by previous findings demonstrating T4’s ability to inhibit reactive oxygen species production by polymorphonuclear leukocytes stimulated with bacterial components (40). Conversely, the pro-inflammatory response induced by the staphylococcal phage A5/80 preparation mirrors the highly disturbed, systemic environment of its host. S. aureus is an extra-intestinal opportunist that predominantly colonizes the anterior nares, respiratory tract, and skin (78), frequently inducing robust systemic inflammatory responses. To successfully reach distant colonization sites of deep tissue, bacteriophages must effectively bypass the epithelial barrier and enter the systemic circulation. This interpretation is supported by previous evidence showing that A5/80 can translocate from the gastrointestinal tract into the bloodstream, a dissemination capacity not observed for T4 (11). This phenotypic divergence likely reflects disparate selective pressures regarding immunogenicity. The primary host of A5/80, *S. aureus*, frequently induces systemic infections and robust inflammatory responses. Consequently, phages targeting such pathogens may be subjected to selective pressures within an already highly activated immune microenvironment. In this context, a pronounced interaction with the mammalian immune system may not incur a fitness cost: rather, the localized induction of inflammation could represent an adaptive trait that increases tissue permeability, thereby facilitating systemic viral dissemination. Although staphylococcal phages can exhibit broad host ranges across diverse species (79), their co-evolution with a major opportunistic pathogen may have selected for a fundamentally distinct immunological footprint compared to gut-resident phages like T4. This strain-specific divergence in immune responses to structurally analogous entities (dsDNA phages) echoes the paradigm of biased receptor signaling well-documented in other cellular pathways. For instance, recent structural biology studies on G protein-coupled receptors (GPCRs), such as GPR4, have demonstrated how subtle extracellular stimuli can translate into highly biased intracellular signaling cascades through specific extracellular structural rearrangements (80).

### 4.3 Translational perspectives: tissue-specific immunity and immune homeostasis

While our study suggests that monocytes act as primary sensors of bacteriophages, it is crucial to interpret these findings within the context of tissue-specific immunity. In a clinical setting, particularly during oral phage therapy, bacteriophages primarily encounter tissue-resident macrophages (e.g., in the intestinal lamina propria) rather than circulating monocytes (81). As previously described, upon entering tissues, circulating monocytes adapt to the local microenvironment, often acquiring specialized, less reactive phenotypes (73). Unlike peripheral blood monocytes, which are poised for rapid pro-inflammatory responses, intestinal macrophages typically exhibit a “tolerogenic” phenotype (inflammation anergy) to prevent excessive reactions to commensal microbiota (82). Therefore, the pronounced cytokine secretion observed in our isolated model unmasks the maximal intrinsic immune potential of these sensors. *In vivo*, this robust signaling is likely buffered by both the tolerogenic tissue microenvironment and broader counter-regulatory immune cross-talk. A parallel adaptation occurs in the dermal compartments, which are important encounter sites during many topical phage applications. While the cutaneous macrophage pool is continuously replenished by circulating blood monocytes, these infiltrating cells undergo stringent local education driven by the dermal microenvironment and skin microbiota. Upon entering the tissue niche, they downregulate their inflammatory potential, adopting specialized phenotypes focused on maintaining barrier homeostasis rather than initiating overt pro-inflammatory cascades (83, 84). This physiological dampening plausibly reconciles the pronounced mechanistic signals captured in our assays with the systemic safety profile consistently reported in clinical practice (69).

This translation from circulating innate immune sensors to tissue-level homeostasis is strongly supported by recent *in vivo* studies. For instance, Gan et al. (85) demonstrated that oral administration of a lytic phage phiW14 targeting *Klebsiella pneumoniae* in a murine model of steatohepatitis not only eradicated the pathogen but also actively shifted the immunological profile within the liver tissue. Specifically, phage therapy downregulated pro-inflammatory MAPK and NF-κB cascades while significantly upregulating the expression of the anti-inflammatory IL-10. This tissue response closely parallels the tolerogenic potential (e.g., IL-10 induction) we observed in our isolated monocyte fraction, reinforcing the concept that phages can interact with the host’s innate immune system to resolve inflammation and restore tissue homeostasis.

### 4.4 Measured and balanced activation: contextualizing the phage-induced immune response

Importantly, the observed phage-mediated response is modest; for context, the pathogenic AdV-5 induced approximately 10-fold higher levels of IFN-γ in our assays. This observation strongly suggests that phages are not recognized by the immune system as a major viral threat, an immunological feature of potential relevance to safety assessments. Beyond its magnitude, the induced immunomodulation is highly specific and balanced. The concurrent secretion of pro-inflammatory (e.g., IFN-γ, IL-2) and regulatory (IL-10) cytokines points to a balanced immunological activity rather than an uncontrolled inflammatory cascade.

Our functional protein-level data build upon and further refine foundational observations regarding this balance. Previously, Van Belleghem et al. (19) demonstrated that exposing bulk human PBMCs to staphylococcal and Pseudomonas phages induced a concurrent transcriptional upregulation of both pro-inflammatory (e.g., IL1B, TNFA, IL6) and anti-inflammatory (e.g., IL10, IL1RN, SOCS3) genes. However, by relying on mixed cell populations and mRNA quantification, the functional translation and exact cellular origins of these signals remained unresolved. By dissecting this response across isolated subpopulations, our study provides functional evidence that this homeostatic signature is not a generic, uniform leukocyte reaction. Instead, it is highly dependent on both the specific phage strain and the targeted immune cell population. For instance, within the total PBMC pool, an effector profile was characteristic of the high-dose A5/80 phage. This functional protein-level outcome strongly corroborates the transcriptomic observations of Borysowski et al., who reported highly significant upregulation of *IL2* gene expression specifically induced by phage A5/80 in the human A549 cell line (10). In that study, our group proposed that this selective induction may be mediated directly by phage virion components, including capsid proteins. The robust activation observed here in our primary cell model extends these findings and highlights the distinctive, immunogenic profile of this staphylococcal phage, which appears to drive a targeted T-cell response clearly exceeding control activity. Conversely, within the isolated monocyte population, a balanced profile (IFN-γ/IL-2/IL-10) was absent for A5/80 but was instead a specific effect of the high-dose T4 phage. This complexity is further highlighted in T helper cells, where the A5/80 phage induced yet another distinct pattern: a co-activation of IFN-γ and the regulatory IL-10, but without a corresponding induction of IL-2.

This balanced induction of inflammatory and regulatory mediators aligns with established innate immune paradigms. For example, Gvirtz et al. (70) observed a comparable simultaneous release of pro-inflammatory cytokines (e.g., IL-1β, TNF-α) and IL-10 in human skin explants, a microenvironment rich in resident professional antigen-presenting cells. This functional parallel with our isolated monocyte fraction suggests a canonical APC response. Consequently, bacteriophages do not appear to function as blunt pro-inflammatory triggers. Instead, they may act as specific modulators that fine-tune the host’s immune network, actively promoting a return to homeostasis (2, 64).

### 4.5 Phage strain-dependent divergence in Th-cell responsiveness

A notable contrast emerged in the adaptive immune response: the A5/80 phage induced a significant response in CD4^+^ cell cultures (such as IL-10), whereas the T4 phage elicited no significant response. This divergence can be hypothesized to stem from the distinct immunological contexts of these two phages, leading to a differential state of host tolerance. Phages similar to T4 are recognized as ubiquitous, symbiotic components of the gut microbiome. According to the Bacteriophage Adherence to Mucus (BAM) model, T4 utilizes its Hoc capsid protein to bind specifically to mucin glycoproteins, effectively sequestering it within the mucus layer. This interpretation is supported by *in vivo* data from Międzybrodzki et al., which demonstrated that under physiological conditions, T4, unlike A5/80, fails to translocate into the bloodstream, even at high doses (11). The authors hypothesize that this is due to T4 being “trapped in the intestinal mucus.” Consequently, circulating T-helper cells from healthy donors are likely systemically tolerized to this non-invasive, commensal particle.

Importantly, non-specific bystander activation alone does not appear to fully account for the specificity of the observed T-helper response. In our preliminary screening, isolated B lymphocytes, despite being professional antigen-presenting cells, did not mount a comparable cytokine response when exposed to the same phage stimuli. This functional divergence likely stems from their distinct mechanisms of antigen uptake. Unlike monocytes, which utilize broad-spectrum macropinocytosis and phagocytosis to readily scavenge large viral particles, resting B cells typically require highly specific B-cell receptor engagement for efficient antigen internalization and subsequent presentation (76). The limited responsiveness observed in those cells may also reflect the considerable functional heterogeneity of human B-cell populations, whose activation states and phenotypic characteristics can be substantially influenced by the surrounding immune milieu and cytokine signals (77). Consequently, the activation of the CD4^+^ axis is likely driven primarily by the potent, non-specific scavenging capacity of monocytes. During this active processing and presentation, monocytes secrete key immunomodulatory signals, such as IL-10, which subsequently shape the downstream T-cell response (48, 73). This functional dichotomy supports the concept that bacteriophage recognition relies on an indirect, phagocyte-dependent interaction rather than universal APC reactivity or direct lymphocyte stimulation.

The consistently low concentrations of IL-17A protein observed in this study, which hovered near the lower limit of quantification, preclude drawing mechanistic conclusions regarding specific Th17 polarization or targeted memory recall. This trace-level secretion should be interpreted primarily through the lens of potential safety assessments. The absence of robust, high-magnitude IL-17A production indicates that exposure to these bacteriophages does not appear to trigger a hyper-inflammatory or pathogenic Th17 cascade (86).

The absence of detectable interleukin-5 (IL-5) secretion was a consistent finding across all experimental conditions, including stimulation with both bacteriophage types and the bacterial component controls. This observation is biologically noteworthy, as IL-5 is a Th2-associated cytokine involved in eosinophil activation and recruitment and is closely linked to Th2-associated inflammation. While it is well-established that bacteriophages are immunogenic and elicit robust specific antibody production *in vivo* (87), the lack of IL-5 coupled with baseline levels of IL-4 indicates that this humoral response is not driven by a Th2-skewed inflammatory cascade. The stimuli evaluated in this study - bacteriophages carrying specific PhAMPs, alongside classical bacterial PAMPs - are canonically known to engage innate and Th1-associated pathways, driven by mediators such as IL-1β and IFN-γ, rather than Th2 cascades. This lack of Th2 activation aligns with results from other experimental models. For instance, Gvirtz et al. (70) also reported IL-5 levels below the limit of detection in a human skin organ culture model stimulated with analogous bacterial PAMPs. While an *ex vivo* assay cannot definitively model clinical hypersensitivity, this lack of Th2-skewed reactivity provides *in vitro* context that aligns with observations that systemic phage administration consistently fails to induce eosinophilia in patients (88). The complex nature of this phage-immune dialogue is consistent with the pleiotropic functions of cytokines; IL-6, for instance, while universally induced in our assays, is known to exert pro-inflammatory and protective properties (89). This supports the growing concept of phages as immune modulators rather than simple activators (2, 66). Crucially, this modulatory capacity is intrinsically dose-dependent. While our data show that increasing the phage dose alters cytokine output, this dynamic is particularly relevant in the clinical context, as phage concentrations *in vivo* are rarely static. They can reach exceptionally high levels locally at the site of infection while remaining low in the systemic circulation (21). This localized “fine-tuning” activity may explain the long-term positive clinical outcomes observed in phage therapy, which have been suggested to persist long after the phages themselves are cleared from the body (21). For instance, Yang et al. (90) reported that intraperitoneal phage administration led to an extreme, localized IL-6 concentration exceeding 62,000 pg/mL in the dialysate, highlighting the profound magnitude of tissue-specific dose responses.

### 4.6 Limitations of the study

Our experimental framework utilized cells isolated exclusively from healthy blood donors. The immunological landscape of patients presenting with active bacterial infections or underlying immune deficiencies is fundamentally different. Their immune cells exist in a pre-activated state, which can substantially modulate their susceptibility to phage-mediated signaling. In fact, previous studies have demonstrated that pre-activation with bacterial antigens renders key immune populations, such as monocytes, largely unresponsive to subsequent phage stimulation, effectively attenuating any additional cytokine production (39). To address this natural biological heterogeneity while optimizing resource allocation, we employed a deliberate, adaptive screening strategy. Initial cohorts (n = 3-4) were analyzed across all subpopulations to establish baseline reactivity. For cell types exhibiting no detectable phage-specific response (e.g., B cells, NK cells, and cytotoxic cells), further recruitment was capped. Conversely, for subpopulations identified as primary responders, specifically monocytes and T helper cells, the cohorts were expanded (n = 7-9) to robustly characterize their distinct cytokine signatures. Thus, while data for non-responding populations represent a targeted screening confirming their lack of reactivity, the key immunomodulatory findings are derived from a representative sample set. Building upon this exploratory, broad-spectrum screening, future studies should aim to dissect the specific cellular crosstalk at a single-cell resolution. While techniques such as targeted synthetic monocyte-T cell co-cultures and intracellular flow cytometry (IC-FACS) are conceptually valuable, their implementation in primary cells is methodologically challenging. The mechanical stress of cell sorting and repetitive washing can inadvertently induce baseline activation artifacts in delicate primary cells (45). Reassuringly, recent high-resolution single-cell studies suggest that innate microbial triggers compartmentalize pro-inflammatory signals predominantly to the monocyte cluster (91). Adapting advanced, minimal-manipulation single-cell technologies will be a critical future direction to definitively map these interactions. Furthermore, the comparative benchmarking with AdV-5 was conducted as a descriptive pilot experiment utilizing PBMCs from a single donor. While it provides contextualization for the magnitude of cytokine secretion, this small sample size limits the broader generalizability of these specific quantitative comparisons.

Important microbiological and methodological variables are linked to the two specific phages investigated, T4 and A5/80. Given the vast genetic and structural diversity of bacteriophages and their associated host-derived microbiological backgrounds, these results cannot be universally extrapolated. Distinct phages possess unique structural proteins and genomic motifs that elicit highly divergent immunological responses; therefore, future studies incorporating *in vivo* models and clinical samples from patient cohorts undergoing phage therapy are necessary to expand upon these findings. Furthermore, we acknowledge that the immunomodulatory profile of the staphylococcal A5/80 phage must be interpreted within the context of its propagation host. Because endotoxin is a pervasive environmental and laboratory contaminant, frequently introduced via water, analytical-grade reagents, and multi-use chromatographic equipment, it represents a universal background inflammatory stimulus in *in vitro* assays (92, 93). Highly enriched primary immune cells are exquisitely sensitive to these trace contaminants, detecting them even in the picogram-to-nanogram per milliliter range, which, if left uncontrolled, can easily mask or misidentify genuine biological effects (94). Therefore, we utilized an *E. coli* LPS control matched to the LAL-quantified endotoxin activity of our final preparations to standardize this unavoidable, process-derived bio-burden. While this approach effectively controls for the Gram-negative environmental background, it does not fully account for host-derived Gram-positive structural components, such as lipoteichoic acid (LTA), bacterial lipoproteins (TLR2 agonists) or peptidoglycan. However, as demonstrated by Kimbrell et al. using LTA, the intrinsic capacity of some Gram-positive components like LTA to induce cytokine secretion is much weaker compared to the highly potent responses elicited by Gram-negative LPS (95). Paradoxically, utilizing commercial Gram-positive cell wall components as standalone controls presents methodological challenges due to the risk of endotoxin contamination (96). Perhaps further research utilizing LTA alongside recombinant phage proteins could isolate the intrinsic immunogenicity of the staphylococcal phage virions from these host-derived variables.

To build upon these observations, future research should address specific, actionable questions. First, to what extent do host-derived Gram-positive bacterial components, such as lipoteichoic acid, that may be present in therapeutic phage preparations contribute to the observed pro-inflammatory responses relative to the intrinsic immunogenicity of purified staphylococcal phage virions? Second, how does the pre-activated immune state of patients presenting with active bacterial infections alter this *ex vivo* cytokine signature during clinical phage therapy?

## 5 Conclusions

This study provides evidence that both model *E. coli* T4 and our therapeutic staphylococcal A5/80 phages are not immunologically inert particles, but rather biologically active entities capable of specific engagement with the human immune system. We observed that these phages elicit a balanced immune response characterized by the co-activation of pro-inflammatory cytokines (such as IFN-γ) alongside the key regulatory mediator IL-10 when engaging a resting immune network. This “homeostatic signature,” combined with the absence of Th2-associated IL-5 secretion and a lower inflammatory potential compared to a pathogenic reference virus (AdV-5), suggests a balanced immunological profile that is highly relevant to the safety assessment of therapeutic bacteriophages. Furthermore, we identified a distinct cell-type specificity within this phage-immune dialogue. In our *ex vivo* model, monocytes emerged as the primary innate sensors orchestrating the response, while T helper cells acted as downstream effectors in a phage strain-dependent manner. In contrast, the remaining lymphoid subpopulations – including cytotoxic T cells, B lymphocytes, and NK cells – did not exhibit direct cytokine responses under phage challenge.

Ultimately, our findings highlight that distinct therapeutic phage preparations—shaped both by their virion characteristics and their specific production backgrounds—can elicit divergent immunomodulatory signatures *ex vivo*. While the gut-resident T4 phage exhibited a tendency toward tolerance, the staphylococcal phage A5/80 preparation triggered a more pronounced pro-inflammatory response. Consequently, future clinical paradigms should view phage therapeutics not merely as inert, targeted bacteriolytic agents, but as complex biological products capable of actively interacting with human immune homeostasis. Acknowledging and standardizing these preparation-specific immunological footprints will be crucial for the safe and effective translation of personalized phage therapies.

## Data Availability

The raw data supporting the conclusions of this article will be made available by the authors, without undue reservation.
