# Supplementary material for: Effect of bacteriophages on cytokine production by human peripheral blood mononuclear cells and their subpopulations
Source: Front Immunol. 2026 Oct 13;17:1916240. doi: 10.3389/fimmu.2026.1916240 (PMC13710000; doi:10.3389/fimmu.2026.1916240)
Supplement: Supplementary file 1 [file DataSheet1.pdf]

Supplementary Material

1 Supplementary Figures and Tables

1.1 Supplementary Figures

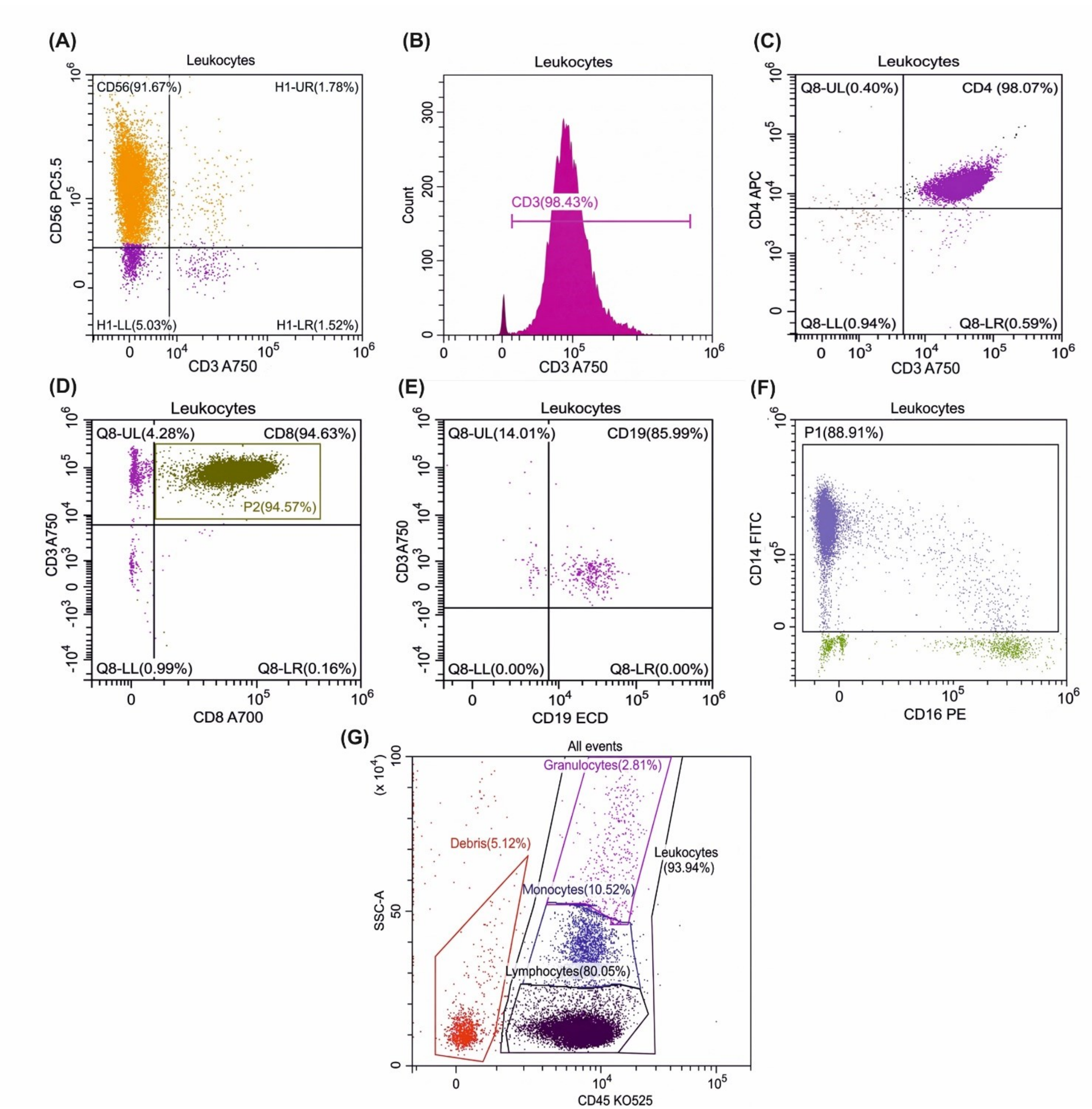

Supplementary Figure S1. Examples of flow cytometry analysis of isolated PBMCs and their populations after subsequent isolation with MojoSort system (BioLegend, San Diego, CA). (A) purity of NK cell population (isolated with MojoSort Human NK Cell Isolation Kit), determined

as percentage of CD56 PC5.5 positive and CD3 A750 negative cells (orange) among $CD45^+$ leukocytes; **(B)** purity of T lymphocyte population (isolated with MojoSort Human CD3 T Cell Isolation Kit) determined as percentage of CD3 A750 positive cells (pink) within the population of $CD45^+$ leukocytes; **(C)** purity of T-helper lymphocyte population (isolated with MojoSort Human CD4 T Cell Isolation Kit) determined as percentage of CD4 APC positive cells (violet) among whole leukocyte population; **(D)** purity of cytotoxic T cell population (isolated with MojoSort Human CD8 T Cell Isolation Kit) determined as percentage of CD8 A700 positive cells (olive green) among whole leukocyte population; **(E)** purity of B lymphocyte population (isolated with MojoSort Human Pan B Cell Isolation Kit) determined as percentage of CD19 ECD positive cells (violet) among whole leukocyte population; **(F)** purity of monocyte population (isolated with MojoSort Human Pan Monocyte Isolation Kit) determined as percentage of CD14 FITC positive/CD16 PE negative and CD14 FITC positive/CD16 PE positive cells (muted violet-blue) among whole leukocyte population; **(G)** dot plot of side scatter (SSC) and CD45 KO525 expression of PBMCs after isolation from buffy coats: light green – monocytes, dark green – granulocytes, dark pink – lymphocytes.

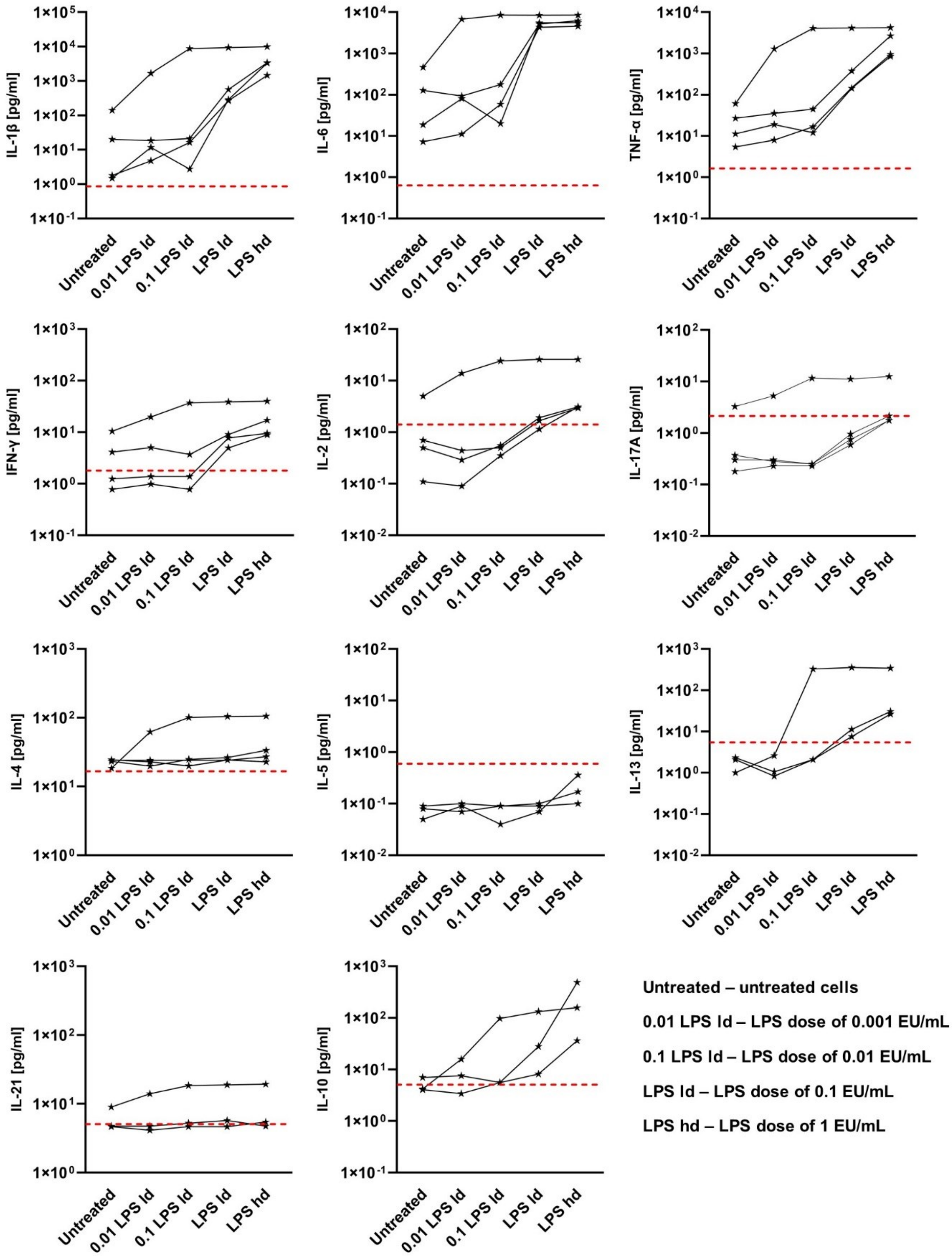

Supplementary Figure S2. Dose-dependent cytokine secretion by PBMCs in response to LPS titration. Data are presented as floating bars spanning from the minimum to the maximum value, with the internal horizontal line representing the median. PBMCs were stimulated for 24 hours with low and high dose of *E. coli* LPS, followed by 10-fold and 100-fold low dose dilutions.

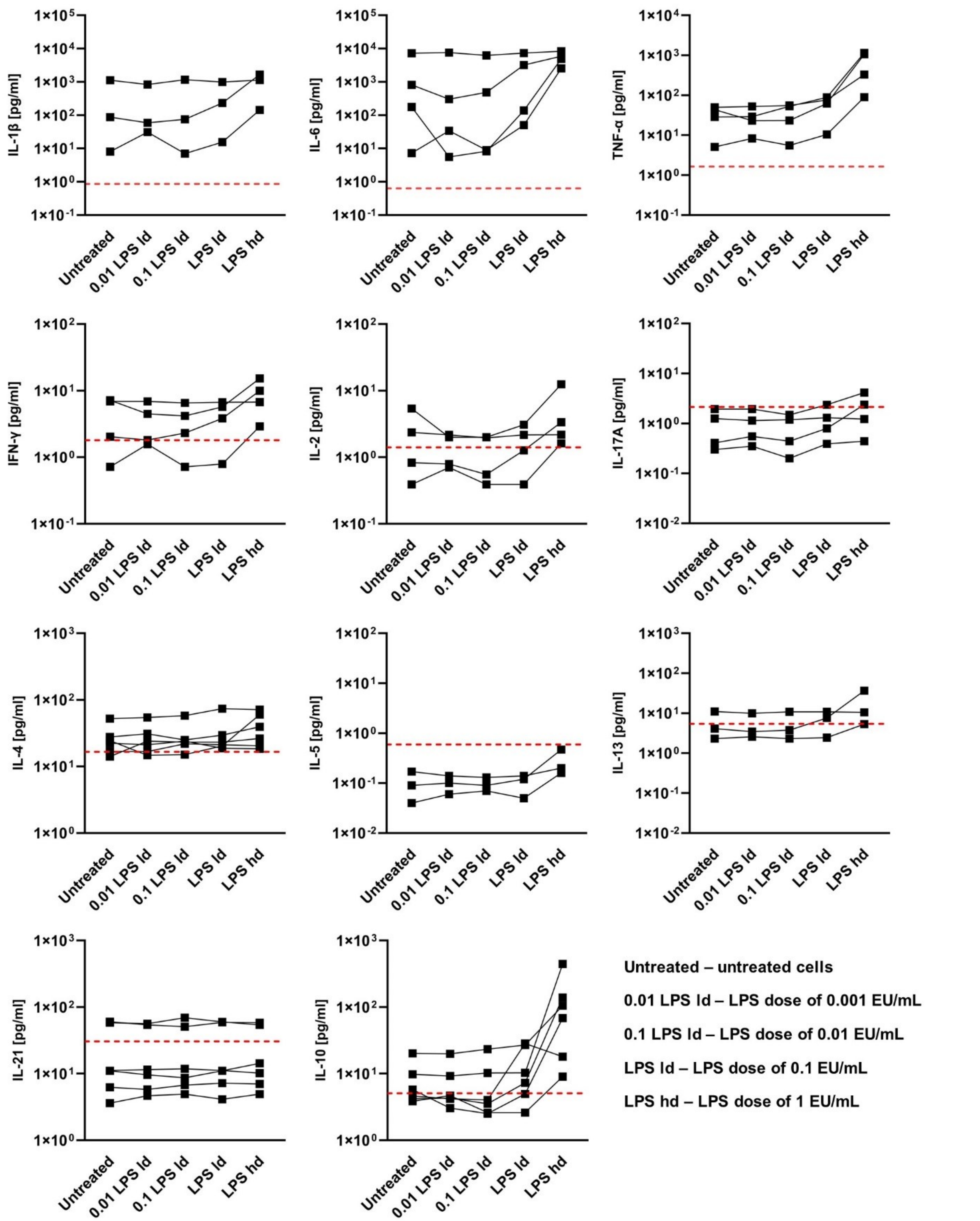

Supplementary Figure S3. Dose-dependent cytokine secretion by monocytes in response to LPS titration. Data are presented as floating bars spanning from the minimum to the maximum value, with the internal horizontal line representing the median. Monocytes were stimulated for 24 hours with low and high dose of *E. coli* LPS, followed by 10-fold and 100-fold low dose dilutions.

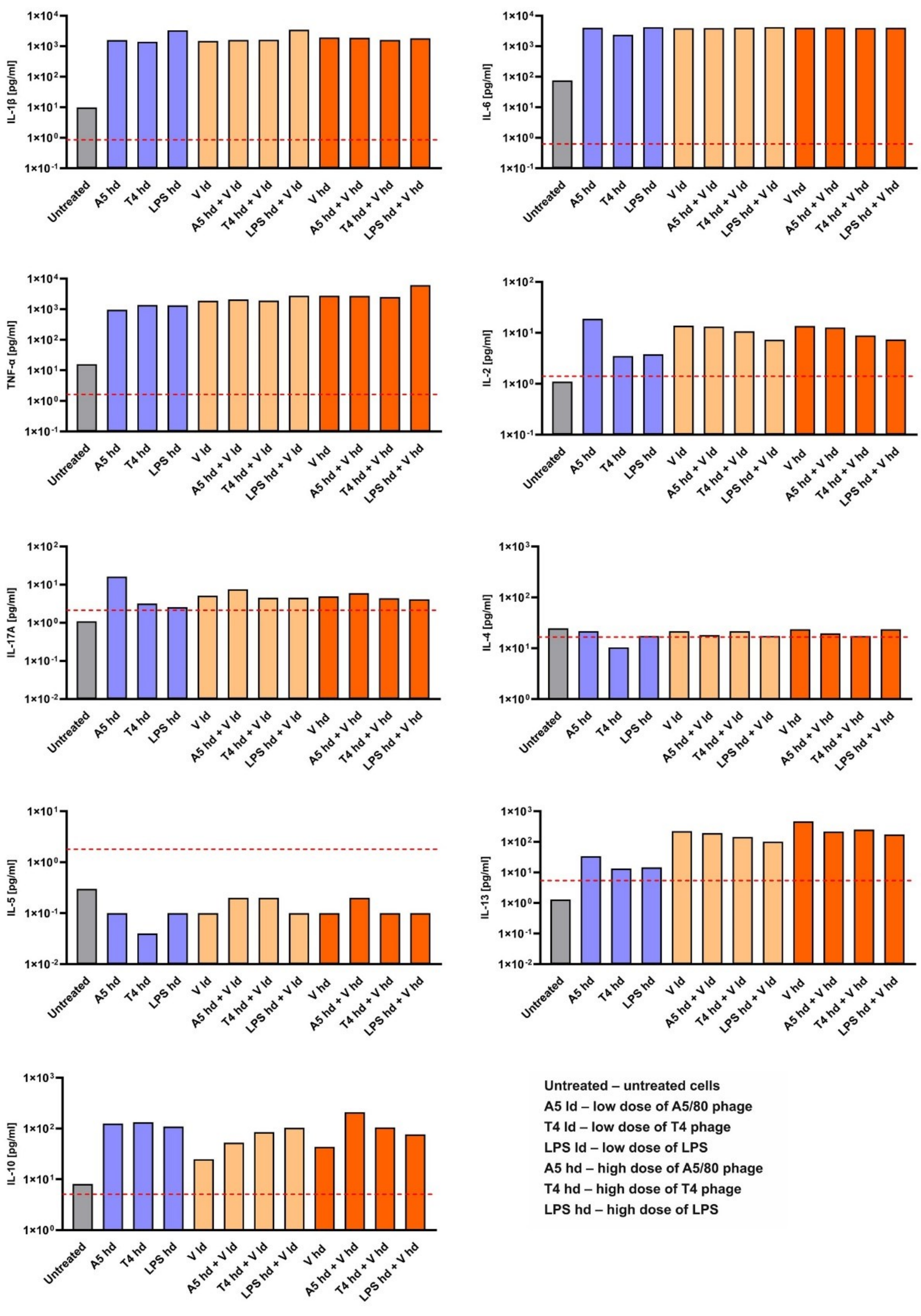

Supplementary Figure S4. Expanded cytokine profiling of PBMCs following exposure to bacteriophages, bacterial components, and AdV-5. Secretion of IL-1β, IL-6, TNF-α, IL-2, IL-17A, IL-4, IL-5, IL-13, and IL-10 by PBMCs in response to high-dose of A5/80 and T4 phages alongside matched control (high dose of *E. coli* LPS) is shown relative to the cell response elicited by a high and low doses of the AdV-5 challenge.

Supplementary Table S1. Comprehensive statistical analysis of cytokine secretion profiles in PBMCs. Data are presented as medians and ranges (minimum–maximum) for each experimental condition. Differences in cytokine concentrations between paired conditions (Condition 1 vs. Condition 2) were evaluated using the two-sided exact Wilcoxon signed-rank test. The resulting exact p-values were subsequently adjusted for multiple comparisons using the Benjamini–Hochberg false discovery rate procedure. Effect sizes are reported as Hodges–Lehmann estimates of the location shift between matched conditions, together with the corresponding 95% confidence intervals. *P*-values < 0.05 and Hodges–Lehmann estimates for which the corresponding 95% confidence interval did not include zero are shown in bold. Abbreviations: CI, confidence interval; n, number of independent biological samples; A5, A5/80 phage; T4, T4 phage; LPS, lipopolysaccharide control; ld, low dose; hd, high dose; –, not applicable.

Cytokine	Condition 1	n	Median 1 (min–max) [pg/mL]	Condition 2	n	Median 2 (min–max) [pg/mL]	Exact p-value	Adjusted p-value	Hodges-Lehmann estimate (95% CI)
**IL-1β**	Untreated	7	1.780 (1.020 – 140.585)	—	—	—	—	—	—
	A5 ld	6	604.891 (179.270 – 9381.698)	LPS ld	6	427.760 (16.818 – 9389.816)	0.6875	0.825	200.220 (-1770.530 – 2927.270)
	T4 ld	6	1193.525 (29.707 – 9771.096)	LPS ld	6	427.760 (16.818 – 9389.816)	0.5625	0.825	177.470 (-420.170 – 3026.870)
	A5 ld	6	604.891 (179.270 – 9381.698)	T4 ld	6	1193.525 (29.707 – 9771.096)	0.5625	0.825	-244.499 (–1350.360 – 711.954)
	A5 hd	7	3364.880 (1588.740 – 12387.026)	LPS hd	7	3341.010 (1449.500 – 9950.377)	0.9375	0.9375	190.375 (-1446.940 – 1711.999)
	T4 hd	7	2381.300 (1400.080 – 4401.959)	LPS hd	7	3341.010 (1449.500 – 9950.377)	0.1563	0.825	-930.923 (-3583.264 – 266.155)
	A5 hd	7	3364.880 (1588.740 – 12387.026)	T4 hd	7	2381.300 (1400.080 – 4401.959)	0.2969	0.825	392.908 (-211.590 – 4223.029)
**IL-6**	Untreated	7	18.770 (1.180 – 461.917)	—	—	—	—	—	—
	A5 ld	6	5681.928 (4021.360 – 8507.868)	LPS ld	6	4746.135 (260.377 – 8493.703)	0.1563	0.9375	657.693 (-592.500 – 5767.579)
	T4 ld	6	4541.980 (210.946 – 8515.123)	LPS ld	6	4746.135 (260.377 – 8493.703)	1	1	-14.006 (-589.550 – 1355.880)
	A5 ld	6	5681.928 (4021.360 – 8507.868)	T4 ld	6	4541.980 (210.946 – 8515.123)	1	1	755.890 (-88.350 – 5817.011)
	A5 hd	7	5216.620 (3325.960 – 8590.940)	LPS hd	7	4570.060 (3460.980 – 8529.871)	0.8125	1	-30.365 (-268.705 – 935.300)
	T4 hd	7	5586.800 (2380.010 – 8247.285)	LPS hd	7	4570.060 (3460.980 – 8529.871)	0.9375	1	-11.885 (-994.620 – 566.360)
	A5 hd	7	5216.620 (3325.960 – 8590.940)	T4 hd	7	5586.800 (2380.010 – 8247.285)	0.5781	1	189.969 (-268.330 – 1006.063)
**TNF-α**	Untreated	7	11.230 (3.210 – 60.966)	—	—	—	—	—	—
	A5 ld	6	371.290 (113.560 – 3763.835)	LPS ld	6	262.785 (14.673 – 4154.121)	0.8438	1	-30.420 (-903.000 – 383.590)
	T4 ld	6	496.585 (20.883 – 3600.581)	LPS ld	6	262.785 (14.673 – 4154.121)	0.8438	1	-6.390 (-553.540 – 393.400)
	A5 ld	6	371.290 (113.560 – 3763.835)	T4 ld	6	496.585 (20.883 – 3600.581)	1	1	26.792 (-550.756 – 267.077)
	A5 hd	7	4238.200 (630.090 – 5053.241)	LPS hd	7	1941.400 (853.300 – 4229.981)	0.2188	0.4375	938.400 (-223.210 – 2655.060)
	T4 hd	7	1361.396 (402.930 – 3284.300)	LPS hd	7	1941.400 (853.300 – 4229.981)	0.2188	0.4375	-433.582 (-1790.124 – 260.195)
	A5 hd	7	4238.200 (630.090 – 5053.241)	T4 hd	7	1361.396 (402.930 – 3284.300)	0.0781	0.4375	1687.286 (-34.778 – 3460.620)
**IFN-γ**	Untreated	7	1.430 (0.780 – 10.411)	—	—	—	—	—	—
	A5 ld	6	8.924 (4.960 – 38.712)	LPS ld	6	7.020 (2.252 – 39.253)	0.4375	0.6563	0.850 (-2.870 – 6.545)
	T4 ld	6	10.075 (1.291 – 39.793)	LPS ld	6	7.020 (2.252 – 39.253)	0.6875	0.825	0.285 (-2.720 – 3.290)
	A5 ld	6	8.924 (4.960 – 38.712)	T4 ld	6	10.075 (1.291 – 39.793)	1	1	-0.150 (-2.710 – 7.506)
	A5 hd	7	33.960 (9.630 – 195.680)	LPS hd	7	17.090 (8.860 – 40.062)	**0.0156**	**0.0469**	**11.758 (1.510 – 96.940)**
	T4 hd	7	16.090 (8.150 – 31.648)	LPS hd	7	17.090 (8.860 – 40.062)	**0.0313**	0.0625	**-0.882 (-4.707 – -0.305)**
	A5 hd	7	33.960 (9.630 – 195.680)	T4 hd	7	16.090 (8.150 – 31.648)	**0.0156**	**0.0469**	**12.781 (1.480 – 97.903)**
**IL-2**	Untreated	7	0.750 (0.110 – 10.850)	—	—	—	—	—	—
	A5 ld	6	3.035 (1.470 – 28.056)	LPS ld	6	1.785 (1.052 – 25.879)	0.0625	0.0938	1.439 (-0.190 – 5.210)
	T4 ld	6	2.730 (1.100 – 27.146)	LPS ld	6	1.785 (1.052 – 25.879)	0.2188	0.2625	0.395 (-0.540 – 1.330)
	A5 ld	6	3.035 (1.470 – 28.056)	T4 ld	6	2.730 (1.100 – 27.146)	0.3125	0.3125	0.840 (-0.750 – 5.750)
	A5 hd	7	8.250 (3.140 – 36.988)	LPS hd	7	3.790 (2.930 – 25.861)	**0.0156**	**0.0469**	**6.742 (0.315 – 17.378)**
	T4 hd	7	3.490 (2.740 – 24.424)	LPS hd	7	3.790 (2.930 – 25.861)	**0.0313**	0.0625	**-0.417 (–1.014 – -0.075)**
	A5 hd	7	8.250 (3.140 – 36.988)	T4 hd	7	3.490 (2.740 – 24.424)	**0.0156**	**0.0469**	**7.351 (0.400 – 18.507)**
**IL-17A**	Untreated	7	0.730 (0.180 – 3.269)	—	—	—	—	—	—
	A5 ld	6	1.390 (0.880 – 19.043)	LPS ld	6	0.850 (0.152 – 11.184)	**0.0313**	0.0938	**3.994 (0.130 – 9.960)**
	T4 ld	6	1.545 (0.301 – 13.151)	LPS ld	6	0.850 (0.152 – 11.184)	0.2188	0.2625	0.210 (-0.810 – 1.967)
	A5 ld	6	1.390 (0.880 – 19.043)	T4 ld	6	1.545 (0.301 – 13.151)	0.2188	0.2625	2.701 (-0.490 – 10.770)
	A5 hd	7	5.060 (2.300 – 17.296)	LPS hd	7	2.610 (1.760 – 12.495)	0.0781	0.1563	3.513 (-0.070 – 9.280)
	T4 hd	7	3.230 (1.650 – 9.871)	LPS hd	7	2.610 (1.760 – 12.495)	0.375	0.375	-0.432 (-1.737 – 0.350)
	A5 hd	7	5.060 (2.300 – 17.296)	T4 hd	7	3.230 (1.650 – 9.871)	**0.0156**	0.0938	**4.601 (0.445 – 10.282)**
**IL-4**	Untreated	7	24.106 (18.438 – 28.510)	—	—	—	—	—	—
	A5 ld	8	23.194 (21.270 – 101.715)	LPS ld	8	24.223 (18.198 – 104.539)	0.8203	0.9844	-0.947 (-3.020 – 2.255)
	T4 ld	8	23.870 (15.985 – 101.142)	LPS ld	8	24.223 (18.198 – 104.539)	0.2031	0.9844	-1.653 (-3.306 – 2.460)
	A5 ld	8	23.194 (21.270 – 101.715)	T4 ld	8	23.870 (15.985 – 101.142)	1	1	-0.077 (-1.840 – 3.216)
	A5 hd	7	24.890 (19.500 – 115.441)	LPS hd	7	23.540 (22.282 – 105.658)	0.7812	0.9844	1.722 (-3.835 – 9.783)
	T4 hd	7	27.111 (19.500 – 86.108)	LPS hd	7	23.540 (22.282 – 105.658)	0.4375	0.9844	-2.255 (-11.415 – 3.754)
	A5 hd	7	24.890 (19.500 – 115.441)	T4 hd	7	27.111 (19.500 – 86.108)	0.625	0.9844	2.130 (-1.640 – 16.086)
**IL-13**	Untreated	6	1.500 (0.510 – 6.890)	—	—	—	—	—	—
	A5 ld	6	10.235 (6.120 – 343.945)	LPS ld	6	9.425 (0.057 – 356.124)	1	1	-0.275 (-12.179 – 9.787)
	T4 ld	6	13.345 (1.053 – 391.266)	LPS ld	6	9.425 (0.057 – 356.124)	0.3125	0.4688	5.395 (-4.400 – 35.142)
	A5 ld	6	10.235 (6.120 – 343.945)	T4 ld	6	13.345 (1.053 – 391.266)	0.4375	0.525	-5.200 (-47.321 – 8.790)
	A5 hd	7	33.060 (18.250 – 566.979)	LPS hd	7	26.420 (20.370 – 343.625)	0.2188	0.4375	4.787 (-2.120 – 174.480)
	T4 hd	7	23.150 (19.400 – 182.512)	LPS hd	7	26.420 (20.370 – 343.625)	0.1094	0.3281	-2.637 (-82.867 – 0.450)
	A5 hd	7	33.060 (18.250 – 566.979)	T4 hd	7	23.150 (19.400 – 182.512)	**0.0313**	0.1875	**6.537 (0.995 – 195.884)**
**IL-21**	Untreated	7	9.003 (4.351 – 103.190)	—	—	—	—	—	—
	A5 ld	8	11.051 (4.272 – 106.920)	LPS ld	8	12.319 (4.670 – 106.980)	**0.0469**	0.1406	**-1.618 (-3.016 – -0.030)**
	T4 ld	8	12.675 (2.236 – 103.670)	LPS ld	8	12.319 (4.670 – 106.980)	0.0781	0.1562	-2.188 (-4.595 – 0.260)
	A5 ld	8	11.051 (4.272 – 106.920)	T4 ld	8	12.675 (2.236 – 103.670)	0.6094	0.6406	0.859 (-0.828 – 2.610)
	A5 hd	7	25.286 (4.400 – 128.870)	LPS hd	7	19.369 (4.351 – 114.460)	**0.0312**	0.1406	**3.861 (1.446 – 9.640)**
	T4 hd	7	15.108 (4.400 – 112.700)	LPS hd	7	19.369 (4.351 – 114.460)	0.6406	0.6406	1.319 (-1.760 – 8.440)
	A5 hd	7	25.286 (4.400 – 128.870)	T4 hd	7	15.108 (4.400 – 112.700)	0.5	0.6406	0.664 (-1.785 – 10.178)
**IL-10**	Untreated	9	4.180 (0.170 – 10.150)	—	—	—	—	—	—
	A5 ld	8	12.130 (4.642 – 87.423)	LPS ld	8	15.155 (2.233 – 132.783)	0.3125	0.5742	-9.460 (-48.340 – 6.930)
	T4 ld	8	20.305 (3.312 – 87.728)	LPS ld	8	15.155 (2.233 – 132.783)	0.6406	0.7688	-3.575 (-29.006 – 9.890)
	A5 ld	8	12.130 (4.642 – 87.423)	T4 ld	8	20.305 (3.312 – 87.728)	0.3828	0.5742	-6.969 (-22.980 – 3.571)
	A5 hd	9	125.379 (22.410 – 855.710)	LPS hd	9	125.078 (23.820 – 489.290)	1	1	-1.410 (-54.910 – 250.405)
	T4 hd	9	50.358 (2.080 – 264.690)	LPS hd	9	125.078 (23.820 – 489.290)	**0.0273**	0.082	**-61.866 (-146.980 – -5.455)**
	A5 hd	9	125.379 (22.410 – 855.710)	T4 hd	9	50.358 (2.080 – 264.690)	**0.0273**	0.082	**65.962 (2.652 – 305.700)**

Supplementary Table S2. Comprehensive statistical analysis of cytokine secretion profiles in monocytes. Data are presented as medians and ranges (minimum–maximum) for each experimental condition. Differences in cytokine concentrations between paired conditions (Condition 1 vs. Condition 2) were evaluated using the two-sided exact Wilcoxon signed-rank test. The resulting exact p-values were subsequently adjusted for multiple comparisons using the Benjamini–Hochberg false discovery rate procedure. Effect sizes are reported as Hodges–Lehmann estimates of the location shift between matched conditions, together with the corresponding 95% confidence intervals. *P*-values < 0.05 and Hodges–Lehmann estimates for which the corresponding 95% confidence interval did not include zero are shown in bold. Abbreviations: CI, confidence interval; n, number of independent biological samples; A5, A5/80 phage; T4, T4 phage; LPS, lipopolysaccharide control; ld, low dose; hd, high dose; –, not applicable.

Cytokine	Condition 1	n	Median 1 (min–max) [pg/mL]	Condition 2	n	Median 2 (min–max) [pg/mL]	Exact p-value	Adjusted p-value	Hodges-Lehmann estimate (95% CI)
**IL-1β**	Untreated	7	8.100 (1.900 – 1125.610)	—	—	—	—	—	—
	A5 ld	7	228.960 (77.310 – 3204.100)	LPS ld	7	95.880 (4.900 – 997.560)	**0.0156**	**0.0313**	**254.895 (79.940 – 1297.800)**
	T4 ld	7	470.310 (48.050 – 3635.580)	LPS ld	7	95.880 (4.900 – 997.560)	**0.0156**	**0.0313**	**421.567 (94.800 – 2635.590)**
	A5 ld	7	228.960 (77.310 – 3204.100)	T4 ld	7	470.310 (48.050 – 3635.580)	**0.0469**	0.0703	**-177.655 (-1613.275 – -9.480)**
	A5 hd	7	2169.310 (209.280 – 5546.710)	LPS hd	7	926.041 (143.240 – 3267.200)	0.0781	0.0938	1466.618 (-67.260 – 3278.960)
	T4 hd	7	2683.850 (1792.100 – 6913.839)	LPS hd	7	926.041 (143.240 – 3267.200)	**0.0156**	**0.0313**	**1882.023 (120.600 – 4111.094)**
	A5 hd	7	2169.310 (209.280 – 5546.710)	T4 hd	7	2683.850 (1792.100 – 6913.839)	0.1563	0.1563	-713.100 (-1834.389 – 252.560)
**IL-6**	Untreated	7	45.710 (7.270 – 7286.560)	—	—	—	—	—	—
	A5 ld	7	3606.250 (368.590 – 9256.710)	LPS ld	7	537.740 (15.610 – 7369.260)	**0.0156**	**0.0469**	**1695.582 (717.124 – 2477.980)**
	T4 ld	7	3498.540 (381.360 – 8097.280)	LPS ld	7	537.740 (15.610 – 7369.260)	**0.0156**	**0.0469**	**1680.341 (502.440 – 2891.310)**
	A5 ld	7	3606.250 (368.590 – 9256.710)	T4 ld	7	3498.540 (381.360 – 8097.280)	0.9375	0.9375	-6.648 (-813.543 – 633.570)
	A5 hd	7	5547.680 (1396.660 – 8371.547)	LPS hd	7	3945.790 (2311.620 – 8371.340)	0.4688	0.7031	394.305 (-1436.140 – 2988.410)
	T4 hd	7	5803.730 (2203.120 – 8051.084)	LPS hd	7	3945.790 (2311.620 – 8371.340)	0.4688	0.7031	803.247 (-1219.805 – 3116.548)
	A5 hd	7	5547.680 (1396.660 – 8371.547)	T4 hd	7	5803.730 (2203.120 – 8051.084)	0.9375	0.9375	-71.170 (-1683.530 – 1081.040)
**TNF-α**	Untreated	7	7.350 (3.220 – 50.060)	—	—	—	—	—	—
	A5 ld	7	138.990 (50.760 – 1162.730)	LPS ld	7	62.337 (9.570 – 267.970)	**0.0156**	**0.0469**	**109.911 (62.670 – 508.482)**
	T4 ld	7	336.100 (38.600 – 1241.020)	LPS ld	7	62.337 (9.570 – 267.970)	**0.0156**	**0.0469**	**295.440 (51.135 – 674.975)**
	A5 ld	7	138.990 (50.760 – 1162.730)	T4 ld	7	336.100 (38.600 – 1241.020)	0.1563	0.2344	-117.200 (-299.411 – 12.160)
	A5 hd	7	1256.210 (269.510 – 4860.350)	LPS hd	7	884.420 (89.180 – 2991.030)	0.375	0.45	698.240 (-769.355 – 2717.821)
	T4 hd	7	859.150 (445.750 – 3853.806)	LPS hd	7	884.420 (89.180 – 2991.030)	0.0781	0.1563	365.667 (-59.270 – 1656.201)
	A5 hd	7	1256.210 (269.510 – 4860.350)	T4 hd	7	859.150 (445.750 – 3853.806)	0.8125	0.8125	-159.515 (-1136.105 – 1850.300)
**IFN-γ**	Untreated	6	1.550 (0.520 – 7.176)	—	—	—	—	—	—
	A5 ld	6	7.077 (1.700 – 10.270)	LPS ld	6	3.915 (0.790 – 6.710)	**0.0313**	0.0938	**2.975 (0.910 – 6.280)**
	T4 ld	6	9.476 (0.720 – 11.070)	LPS ld	6	3.915 (0.790 – 6.710)	0.0625	0.125	4.700 (–0.070 – 9.440)
	A5 ld	6	7.077 (1.700 – 10.270)	T4 ld	6	9.476 (0.720 – 11.070)	0.2188	0.2625	-1.645 (-4.400 – 0.980)
	A5 hd	7	10.120 (3.910 – 33.285)	LPS hd	7	6.890 (2.910 – 19.770)	0.4688	0.4688	2.975 (-4.670 – 11.813)
	T4 hd	7	9.860 (9.420 – 34.918)	LPS hd	7	6.890 (2.910 – 19.770)	**0.0313**	0.0938	**3.838 (1.275 – 11.975)**
	A5 hd	7	10.120 (3.910 – 33.285)	T4 hd	7	9.860 (9.420 – 34.918)	0.2188	0.2625	-2.111 (-8.340 – 2.115)
**IL-2**	Untreated	7	0.830 (0.240 – 5.398)	—	—	—	—	—	—
	A5 ld	7	2.060 (0.590 – 4.990)	LPS ld	7	1.270 (0.200 – 3.079)	**0.0156**	**0.0469**	**1.075 (0.390 – 1.631)**
	T4 ld	7	2.770 (0.500 – 6.215)	LPS ld	7	1.270 (0.200 – 3.079)	**0.0156**	**0.0469**	**1.230 (0.330 – 2.408)**
	A5 ld	7	2.060 (0.590 – 4.990)	T4 ld	7	2.770 (0.500 – 6.215)	0.5781	0.5781	-0.323 (-1.160 – 0.475)
	A5 hd	7	3.480 (1.980 – 21.125)	LPS hd	7	2.180 (1.470 – 12.502)	**0.0469**	0.0703	**1.005 (0.120 – 4.881)**
	T4 hd	7	3.110 (2.670 – 22.591)	LPS hd	7	2.180 (1.470 – 12.502)	**0.0313**	0.0625	**1.212 (0.515 – 5.745)**
	A5 hd	7	3.480 (1.980 – 21.125)	T4 hd	7	3.110 (2.670 – 22.591)	0.2969	0.3563	-0.418 (-1.078 – 0.310)
**IL-17A**	Untreated	6	0.360 (0.140 – 1.935)	—	—	—	—	—	—
	A5 ld	7	1.150 (0.290 – 3.270)	LPS ld	7	0.800 (0.050 – 2.382)	**0.0312**	0.0625	**0.556 (0.175 – 1.050)**
	T4 ld	7	1.680 (0.340 – 3.713)	LPS ld	7	0.800 (0.050 – 2.382)	**0.0313**	0.0625	**0.772 (0.190 – 1.206)**
	A5 ld	7	1.150 (0.290 – 3.270)	T4 ld	7	1.680 (0.340 – 3.713)	0.1875	0.1875	-0.171 (-0.490 – 0.060)
	A5 hd	7	1.690 (1.160 – 9.652)	LPS hd	7	1.230 (0.440 – 4.154)	0.1563	0.1875	0.552 (-0.235 – 3.059)
	T4 hd	7	1.960 (1.470 – 9.871)	LPS hd	7	1.230 (0.440 – 4.154)	**0.0313**	0.0625	**0.830 (0.135 – 3.404)**
	A5 hd	7	1.690 (1.160 – 9.652)	T4 hd	7	1.960 (1.470 – 9.871)	0.1563	0.1875	-0.305 (-0.730 – 0.130)
**IL-4**	Untreated	9	22.440 (14.050 – 52.300)	—	—	—	—	—	—
	A5 ld	7	26.400 (13.620 – 63.390)	LPS ld	7	21.140 (12.980 – 74.100)	0.625	1	0.757 (-5.035 – 5.973)
	T4 ld	7	20.930 (15.700 – 54.460)	LPS ld	7	21.140 (12.980 – 74.100)	0.9375	1	1.175 (-11.200 – 12.014)
	A5 ld	7	26.400 (13.620 – 63.390)	T4 ld	7	20.930 (15.700 – 54.460)	1	1	-0.445 (-7.276 – 6.720)
	A5 hd	9	26.000 (11.400 – 95.281)	LPS hd	9	20.470 (11.400 – 71.540)	0.1641	0.4922	5.310 (-2.665 – 18.614)
	T4 hd	9	26.400 (12.980 – 98.826)	LPS hd	9	20.470 (11.400 – 71.540)	**0.0156**	0.0938	**4.655 (1.205 – 20.501)**
	A5 hd	9	26.000 (11.400 – 95.281)	T4 hd	9	26.400 (12.980 – 98.826)	0.8203	1	-0.655 (-9.050 – 8.375)
**IL-13**	Untreated	7	2.320 (0.000 – 11.040)	—	—	—	—	—	—
	A5 ld	7	9.870 (0.000 – 12.710)	LPS ld	7	3.310 (0.000 – 10.810)	**0.0312**	0.0938	**3.100 (0.985 – 5.520)**
	T4 ld	7	12.560 (0.000 – 24.270)	LPS ld	7	3.310 (0.000 – 10.810)	**0.0312**	0.0938	**4.668 (1.680 – 10.795)**
	A5 ld	7	9.870 (0.000 – 12.710)	T4 ld	7	12.560 (0.000 – 24.270)	0.1875	0.225	-1.433 (-6.645 – 0.360)
	A5 hd	7	17.430 (9.370 – 225.642)	LPS hd	7	9.370 (0.000 – 36.670)	0.125	0.1875	5.817 (-0.840 – 117.471)
	T4 hd	7	23.080 (11.520 – 270.074)	LPS hd	7	9.370 (0.000 – 36.670)	0.0781	0.1563	8.375 (-0.320 – 140.367)
	A5 hd	7	17.430 (9.370 – 225.642)	T4 hd	7	23.080 (11.520 – 270.074)	0.2969	0.2969	-3.642 (-25.116 – 3.810)
**IL-21**	Untreated	6	11.121 (3.610 – 60.400)	—	—	—	—	—	—
	A5 ld	6	16.876 (4.670 – 58.580)	LPS ld	6	11.121 (4.140 – 60.040)	1	1	0.115 (-5.580 – 11.040)
	T4 ld	6	11.335 (3.480 – 61.230)	LPS ld	6	11.121 (4.140 – 60.040)	0.8438	1	-0.450 (-2.370 – 1.409)
	A5 ld	6	16.876 (4.670 – 58.580)	T4 ld	6	11.335 (3.480 – 61.230)	0.625	1	0.595 (-3.854 – 12.020)
	A5 hd	6	15.374 (4.930 – 63.580)	LPS hd	6	12.264 (4.930 – 58.420)	0.25	1	1.925 (-0.655 – 6.078)
	T4 hd	6	15.955 (4.140 – 61.310)	LPS hd	6	12.264 (4.930 – 58.420)	0.5625	1	0.585 (-5.880 – 7.050)
	A5 hd	6	15.374 (4.930 – 63.580)	T4 hd	6	15.955 (4.140 – 61.310)	1	1	0.299 (-8.360 – 11.040)
**IL-10**	Untreated	9	5.767 (3.860 – 20.250)	—	—	—	—	—	—
	A5 ld	9	14.380 (4.040 – 106.850)	LPS ld	9	12.610 (2.610 – 41.330)	0.1641	0.1969	7.810 (-6.185 – 34.775)
	T4 ld	9	16.720 (4.240 – 183.000)	LPS ld	9	12.610 (2.610 – 41.330)	0.0547	0.1641	17.445 (-4.405 – 73.790)
	A5 ld	9	14.380 (4.040 – 106.850)	T4 ld	9	16.720 (4.240 – 183.000)	0.0977	0.1934	-10.805 (-40.753 – 12.120)
	A5 hd	9	161.790 (24.080 – 687.850)	LPS hd	9	68.683 (9.050 – 1040.730)	0.1289	0.1934	91.160 (-140.685 – 312.599)
	T4 hd	9	200.140 (8.150 – 1267.100)	LPS hd	9	68.683 (9.050 – 1040.730)	**0.0078**	**0.0469**	**118.450 (21.750 – 339.595)**
	A5 hd	9	161.790 (24.080 – 687.850)	T4 hd	9	200.140 (8.150 – 1267.100)	0.4961	0.4961	-14.505 (-336.710 – 68.700)

Supplementary Table S3. Comprehensive statistical analysis of cytokine secretion profiles in T helper cells. Data are presented as medians and ranges (minimum–maximum) for each experimental condition. Differences in cytokine concentrations between paired conditions (Condition 1 vs. Condition 2) were evaluated using the two-sided exact Wilcoxon signed-rank test. The resulting exact p-values were subsequently adjusted for multiple comparisons using the Benjamini–Hochberg false discovery rate procedure. Effect sizes are reported as Hodges–Lehmann estimates of the location shift between matched conditions, together with the corresponding 95% confidence intervals. *P*-values < 0.05 and Hodges–Lehmann estimates for which the corresponding 95% confidence interval did not include zero are shown in bold. Abbreviations: CI, confidence interval; n, number of independent biological samples; A5, A5/80 phage; T4, T4 phage; LPS, lipopolysaccharide control; ld, low dose; hd, high dose; –, not applicable.

Cytokine	Condition 1	n	Median 1 (min–max) [pg/mL]	Condition 2	n	Median 2 (min–max) [pg/mL]	Exact p-value	Adjusted p-value	Hodges-Lehmann estimate (95% CI)
**IL-1β**	Untreated	7	0.500 (0.050 – 20.810)	—	—	—	—	—	—
	A5 ld	7	17.690 (0.580 – 225.000)	LPS ld	7	29.180 (0.430 – 275.420)	0.8125	0.9375	-4.980 (-125.280 – 57.885)
	T4 ld	7	27.010 (0.170 – 272.020)	LPS ld	7	29.180 (0.430 – 275.420)	0.6875	0.9375	11.236 (-124.335 – 99.670)
	A5 ld	7	17.690 (0.580 – 225.000)	T4 ld	7	27.010 (0.170 – 272.020)	0.2969	0.5938	-11.192 (-61.860 – 6.805)
	A5 hd	7	133.590 (1.690 – 4146.760)	LPS hd	7	56.480 (1.390 – 1582.220)	0.1563	0.4688	78.925 (-7.710 – 2564.540)
	T4 hd	7	118.090 (0.400 – 2440.917)	LPS hd	7	56.480 (1.390 – 1582.220)	0.9375	0.9375	-1.880 (-508.830 – 1079.361)
	A5 hd	7	133.590 (1.690 – 4146.760)	T4 hd	7	118.090 (0.400 – 2440.917)	0.0781	0.4688	274.475 (-5.965 – 1965.975)
**IL-6**	Untreated	7	0.940 (0.320 – 250.600)	—	—	—	—	—	—
	A5 ld	7	98.910 (11.600 – 3186.610)	LPS ld	7	115.867 (5.420 – 5853.770)	0.9375	1	-17.782 (-2726.970 – 905.947)
	T4 ld	7	531.300 (6.250 – 3248.960)	LPS ld	7	115.867 (5.420 – 5853.770)	0.5781	0.8672	328.567 (-2660.820 – 990.087)
	A5 ld	7	98.910 (11.600 – 3186.610)	T4 ld	7	531.300 (6.250 – 3248.960)	0.2969	0.5938	-35.742 (-419.100 – 240.720)
	A5 hd	7	979.570 (26.450 – 7946.382)	LPS hd	7	597.900 (11.570 – 7871.414)	0.2969	0.5938	46.252 (-467.750 – 228.319)
	T4 hd	7	884.720 (11.920 – 8454.980)	LPS hd	7	597.900 (11.570 – 7871.414)	0.1094	0.5938	153.870 (-118.459 – 547.510)
	A5 hd	7	979.570 (26.450 – 7946.382)	T4 hd	7	884.720 (11.920 – 8454.980)	1	1	-1.360 (-894.225 – 179.733)
**TNF-α**	Untreated	7	2.500 (0.130 – 71.070)	—	—	—	—	—	—
	A5 ld	7	37.994 (1.960 – 916.890)	LPS ld	7	12.450 (0.630 – 297.620)	1	1	-0.282 (-121.435 – 363.095)
	T4 ld	7	26.073 (0.910 – 385.560)	LPS ld	7	12.450 (0.630 – 297.620)	0.9375	1	-0.637 (-110.745 – 97.450)
	A5 ld	7	37.994 (1.960 – 916.890)	T4 ld	7	26.073 (0.910 – 385.560)	0.6875	1	4.438 (-10.510 – 271.625)
	A5 hd	7	94.880 (3.290 – 2729.180)	LPS hd	7	61.490 (1.930 – 1116.850)	0.1094	0.3281	25.382 (-14.710 – 822.860)
	T4 hd	7	84.630 (1.850 – 1120.710)	LPS hd	7	61.490 (1.930 – 1116.850)	0.0781	0.3281	18.320 (-0.010 – 148.127)
	A5 hd	7	94.880 (3.290 – 2729.180)	T4 hd	7	84.630 (1.850 – 1120.710)	0.375	0.75	8.047 (-81.210 – 809.360)
**IFN-γ**	Untreated	7	1.300 (0.170 – 8.990)	—	—	—	—	—	—
	A5 ld	7	3.250 (0.350 – 1077.550)	LPS ld	7	4.304 (0.490 – 687.090)	1	1	-0.043 (-2.060 – 195.385)
	T4 ld	7	5.660 (0.370 – 768.440)	LPS ld	7	4.304 (0.490 – 687.090)	0.4688	0.6938	0.841 (-2.900 – 41.353)
	A5 ld	7	3.250 (0.350 – 1077.550)	T4 ld	7	5.660 (0.370 – 768.440)	0.4688	0.6938	0.425 (-5.155 – 154.791)
	A5 hd	7	18.760 (0.670 – 6610.930)	LPS hd	7	6.210 (0.440 – 1060.060)	**0.0469**	0.2812	**12.166 (0.200 – 2785.420)**
	T4 hd	7	11.870 (0.910 – 1028.730)	LPS hd	7	6.210 (0.440 – 1060.060)	0.3438	0.6938	2.947 (-15.320 – 12.085)
	A5 hd	7	18.760 (0.670 – 6610.930)	T4 hd	7	11.870 (0.910 – 1028.730)	0.5781	0.6938	7.145 (-8.205 – 2798.255)
**IL-2**	Untreated	7	0.690 (0.110 – 9.580)	—	—	—	—	—	—
	A5 ld	7	1.077 (0.120 – 36.820)	LPS ld	7	0.750 (0.240 – 28.800)	0.6875	0.6875	-0.138 (-1.880 – 4.065)
	T4 ld	7	1.524 (0.110 – 28.100)	LPS ld	7	0.750 (0.240 – 28.800)	0.2969	0.5938	-0.148 (-0.490 – 0.352)
	A5 ld	7	1.077 (0.120 – 36.820)	T4 ld	7	1.524 (0.110 – 28.100)	0.5781	0.6875	-0.133 (-1.775 – 4.305)
	A5 hd	7	2.690 (0.350 – 86.310)	LPS hd	7	1.835 (0.350 – 28.840)	0.0938	0.2812	3.787 (0.000 – 31.679)
	T4 hd	7	4.252 (0.350 – 33.770)	LPS hd	7	1.835 (0.350 – 28.840)	0.0938	0.2812	1.987 (0.000 – 6.099)
	A5 hd	7	2.690 (0.350 – 86.310)	T4 hd	7	4.252 (0.350 – 33.770)	0.4062	0.6094	2.375 (-4.085 – 28.006)
**IL-17A**	Untreated	7	0.200 (0.090 – 1.760)	—	—	—	—	—	—
	A5 ld	7	0.630 (0.060 – 11.340)	LPS ld	7	0.270 (0.130 – 7.550)	0.875	0.875	0.010 (-0.315 – 1.915)
	T4 ld	7	0.582 (0.130 – 11.470)	LPS ld	7	0.270 (0.130 – 7.550)	0.4062	0.5625	0.244 (-0.220 – 2.116)
	A5 ld	7	0.630 (0.060 – 11.340)	T4 ld	7	0.582 (0.130 – 11.470)	0.1563	0.3125	-0.090 (-0.700 – 0.064)
	A5 hd	7	1.910 (0.130 – 24.750)	LPS hd	7	0.743 (0.130 – 10.270)	**0.0469**	0.2813	**2.400 (0.010 – 9.463)**
	T4 hd	7	1.719 (0.290 – 10.100)	LPS hd	7	0.743 (0.130 – 10.270)	0.1563	0.3125	0.679 (-0.065 – 3.943)
	A5 hd	7	1.910 (0.130 – 24.750)	T4 hd	7	1.719 (0.290 – 10.100)	0.4688	0.5625	1.650 (-3.295 – 8.950)
**IL-4**	Untreated	6	20.959 (11.970 – 27.130)	—	—	—	—	—	—
	A5 ld	6	19.841 (8.142 – 29.210)	LPS ld	6	18.934 (12.434 – 25.608)	0.8438	0.8438	0.907 (-4.292 – 9.240)
	T4 ld	6	17.049 (12.434 – 27.111)	LPS ld	6	18.934 (12.434 – 25.608)	0.5	0.8438	1.502 (-3.618 – 4.880)
	A5 ld	6	19.841 (8.142 – 29.210)	T4 ld	6	17.049 (12.434 – 27.111)	0.6875	0.8438	0.678 (-6.998 – 4.966)
	A5 hd	6	20.851 (8.142 – 36.497)	LPS hd	6	19.782 (11.970 – 29.547)	0.8438	0.8438	0.680 (-7.265 – 7.966)
	T4 hd	6	22.008 (9.730 – 35.130)	LPS hd	6	19.782 (11.970 – 29.547)	0.3125	0.8438	2.648 (-3.511 – 8.000)
	A5 hd	6	20.851 (8.142 – 36.497)	T4 hd	6	22.008 (9.730 – 35.130)	0.8438	0.8438	-1.719 (-10.800 – 5.194)
**IL-13**	Untreated	7	0.930 (0.426 – 2.610)	—	—	—	—	—	—
	A5 ld	7	2.926 (0.250 – 17.350)	LPS ld	7	1.394 (0.330 – 13.230)	0.9375	0.9375	–0.098 (–2.205 – 2.145)
	T4 ld	7	2.440 (0.510 – 18.270)	LPS ld	7	1.394 (0.330 – 13.230)	0.5781	0.6938	0.522 (–2.310 – 3.160)
	A5 ld	7	2.926 (0.250 – 17.350)	T4 ld	7	2.440 (0.510 – 18.270)	0.4688	0.6938	–0.285 (–1.595 – 0.426)
	A5 hd	7	6.910 (0.830 – 46.320)	LPS hd	7	3.910 (0.500 – 21.870)	0.0781	0.4688	2.520 (–0.140 – 13.725)
	T4 hd	7	5.630 (1.290 – 19.320)	LPS hd	7	3.910 (0.500 – 21.870)	0.2188	0.5938	0.670 (–1.215 – 4.580)
	A5 hd	7	6.910 (0.830 – 46.320)	T4 hd	7	5.630 (1.290 – 19.320)	0.2969	0.5938	1.073 (–0.315 – 14.140)
**IL-21**	Untreated	6	3.927 (3.006 – 6.272)	—	—	—	—	—	—
	A5 ld	6	4.349 (3.006 – 5.040)	LPS ld	6	3.725 (1.874 – 5.482)	0.2188	0.5625	0.480 (-0.483 – 1.386)
	T4 ld	6	4.044 (2.122 – 4.999)	LPS ld	6	3.725 (1.874 – 5.482)	0.6875	0.825	0.248 (-0.530 – 1.030)
	A5 ld	6	4.349 (3.006 – 5.040)	T4 ld	6	4.044 (2.122 – 4.999)	0.375	0.5625	0.268 (-0.127 – 1.138)
	A5 hd	6	4.441 (2.880 – 5.482)	LPS hd	6	3.805 (2.122 – 5.800)	0.375	0.5625	0.500 (-0.724 – 1.590)
	T4 hd	6	4.820 (1.870 – 5.880)	LPS hd	6	3.805 (2.122 – 5.800)	0.375	0.5625	0.534 (-0.132 – 1.350)
	A5 hd	6	4.441 (2.880 – 5.482)	T4 hd	6	4.820 (1.870 – 5.880)	1	1	–0.165 (-1.437 – 1.019)
**IL-10**	Untreated	7	10.036 (2.427 – 16.670)	—	—	—	—	—	—
	A5 ld	7	8.371 (3.255 – 111.960)	LPS ld	7	9.596 (3.292 – 46.840)	0.8125	0.9375	-0.032 (-1.883 – 32.545)
	T4 ld	7	12.359 (3.098 – 87.640)	LPS ld	7	9.596 (3.292 – 46.840)	0.1563	0.375	2.117 (-0.926 – 21.781)
	A5 ld	7	8.371 (3.255 – 111.960)	T4 ld	7	12.359 (3.098 – 87.640)	0.9375	0.9375	0.209 (-6.020 – 12.875)
	A5 hd	7	19.280 (6.916 – 353.300)	LPS hd	7	9.749 (4.721 – 165.450)	**0.0469**	0.2813	**14.673 (0.170 – 101.565)**
	T4 hd	7	13.170 (6.916 – 66.400)	LPS hd	7	9.749 (4.721 – 165.450)	0.5781	0.8672	1.697 (-50.435 – 29.130)
	A5 hd	7	19.280 (6.916 – 353.300)	T4 hd	7	13.170 (6.916 – 66.400)	0.1875	0.375	5.995 (-4.952 – 147.960)
